# Positive Feedback Loop of Histone Lactylation‐Driven HNRNPC Promotes Autophagy to Confer Pancreatic Ductal Adenocarcinoma Gemcitabine Resistance

**DOI:** 10.1002/advs.202510483

**Published:** 2025-11-27

**Authors:** Xi‐Tai Huang, Jiao Chen, En‐Liang Zhu, Ming‐Jian Ma, Yang‐Yin‐Hui Yu, Ying‐Qin Zhu, Jing‐Yuan Ye, Jin‐Zhao Xie, Zi‐Yi Zhao, Xiao‐Yu Yin

**Affiliations:** ^1^ Department of Pancreato‐Biliary Surgery The First Affiliated Hospital of Sun Yat‐sen University Guangzhou 510080 China

**Keywords:** autophagy, chemoresistance, histone lactylation, HNRNPC, N6‐methyladenosine

## Abstract

Gemcitabine resistance remains a primary determinant of poor survival outcomes in pancreatic ductal adenocarcinoma (PDAC) patients, underscoring the urgent need to elucidate its molecular mechanisms and develop effective countermeasures. Here, gemcitabine‐resistant pancreatic cancer cell lines and patient‐derived xenograft (PDX) models are established, followed by high‐throughput sequencing, which identified heterogeneous nuclear ribonucleoprotein C (HNRNPC) as a significantly upregulated factor in chemoresistant tumors. Silencing of HNRNPC expression substantially restores sensitivity to gemcitabine treatment in vitro and vivo. Mechanistically, multi‐omics analysis reveals that histone H3 lysine18 lactylation (H3K18la) drives HNRNPC overexpression. HNRNPC stabilizes TNF receptor‐associated factor 6 (TRAF6) transcripts in an N6‐methyladenosine(m6A) ‐dependent manner, thereby activating autophagy to mediate gemcitabine resistance. Concurrently, HNRNPC orchestrates a metabolic reprogramming cascade by similarly stabilizing aldehyde dehydrogenase 1 family member A3 (ALDH1A3) mRNA, which enhances glycolysis and H3K18la levels, establishing a self‐reinforcing histone lactylation‐HNRNPC positive feedback loop. Notably, pharmacological inhibition of ALDH1A3 using 673A effectively disrupted this regulatory circuit and exerts a synergistic effect with gemcitabine in PDX. These findings not only delineate a histone lactylation‐driven positive feedback loop sustaining chemoresistance through HNRNPC‐mediated autophagy activation, but also develop the potential of 673A as a promising clinical candidate for overcoming gemcitabine resistance in PDAC treatment.

## Graphical abstract

Histone 3 lysine18 lactylation (H3K18la) drives heterogeneous nuclear ribonucleoprotein C (HNRNPC) overexpression, activating autophagy to mediate gemcitabine resistance by stabilizing TNF receptor‐associated factor 6 (TRAF6) mRNA. Concurrently, HNRNPC stabilizes aldehyde dehydrogenase 1 family member A3 (ALDH1A3) mRNA, which enhances glycolysis and H3K18la levels, establishing positive feedback loop. 673A disrupts this regulatory circuit and synergizes with gemcitabine.

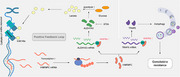

## 1 Introduction

Pancreatic ductal adenocarcinoma (PDAC) represents one of the most lethal malignancies in oncology, characterized by an exceptionally poor prognosis with a five‐year survival rate below 13%.^[^ ^1^ ^]^ Clinical management is further complicated by the fact that ≈80% of patients present with locally advanced or metastatic disease at diagnosis, rendering surgical intervention unfeasible in most cases.^[^ ^2^ ^]^ Current therapeutic paradigms for advanced PDAC continue to rely on gemcitabine‐based combination chemotherapy regimens as the cornerstone of systemic treatment.^[^ ^3^ ^]^ While a subset of patients demonstrates initial clinical response to gemcitabine therapy, the clinical benefit is invariably limited by the rapid emergence of chemoresistance, which significantly compromises treatment outcomes.^[^ ^4^ ^]^ These clinical challenges underscore the critical need to develop innovative strategies for enhancing chemosensitivity to gemcitabine and elucidating the molecular mechanisms underlying treatment resistance, thereby identifying novel therapeutic targets to improve clinical outcomes in PDAC management.

N6‐methyladenosine (m6A), the most prevalent internal mRNA modification in eukaryotic systems, serves as a pivotal epigenetic mechanism governing post‐transcriptional regulation.^[^ ^5^ ^]^ This dynamic modification is enzymatically orchestrated through the opposing activities of writer complexes (methyltransferases) and eraser proteins (demethylases), establishing a sophisticated regulatory equilibrium.^[^ ^6^ ^]^ The biological consequences of m6A modification are functionally executed by specialized “reader” proteins that decode methylation patterns through selective binding, thereby directing spatiotemporal control of RNA metabolism, including stability, splicing, and translational efficiency.^[^ ^7^ ^]^ Emerging evidence has revealed that m6A methylation plays a critical regulatory role in tumorigenesis and progression of various malignancies, particularly in mediating chemoresistance pathways through its dynamic control of cancer‐related transcriptomes.^[^ ^8^ ^]^ Heterogeneous nuclear ribonucleoprotein C (HNRNPC), a subfamily of heterogeneous nuclear ribonucleoproteins (hnRNPs), functionally participates in RNA metabolism processes through their involvement in splicing regulation, mRNA stabilization, and nucleocytoplasmic trafficking mechanisms by binding to mRNA.^[^ ^9^, ^10^, ^11^ ^]^ Accumulating evidence reveals HNRNPC's pivotal role in diverse human cancers. For instance, HNRNPC stabilizes IRAK1 mRNA through m6A‐mediated post‐transcriptional regulation, activating the MAPK signaling cascade to drive glioma progression.^[^ ^12^ ^]^ Moreover, elevated HNRNPC expression is significantly associated with diminished therapeutic efficacy of cisplatin‐based chemotherapy in lung cancer patients.^[^ ^13^ ^]^ In our recent study, we discovered that HNRNPC expression is upregulated in gemcitabine‐resistant PDAC. Therefore, we hypothesize that HNRNPC may also play a critical role in gemcitabine resistance in PDAC.

Histone lactylation, a newly discovered post‐translational modification, has been demonstrated to regulate gene expression.^[^ ^14^ ^]^ This modification predominantly occurs on lysine residues, particularly at histone H3 lysine 18 (H3K18la).^[^ ^15^ ^]^ Accumulating evidence indicates that H3K18la plays significant roles in various malignancies through transcriptional regulation. In gastric cancer (GC), studies have revealed that elevated H3K18la levels are associated with poor clinical prognosis.^[^ ^16^ ^]^ Furthermore, research in hepatocellular carcinoma (HCC) has shown that H3K18la activates PD‐L1 expression, thereby mediating tumor immune escape.^[^ ^17^ ^]^ However, the role of histone lactylation in PDAC gemcitabine resistance remains to be clarified.

Building on these findings, the present study was designed to elucidate whether HNRNPC contributes to gemcitabine resistance in PDAC. Our analyses revealed a consistent upregulation of HNRNPC expression in gemcitabine‐resistant PDAC cells, patient‐derived xenograft (PDX) models, and clinical specimens, with elevated HNRNPC levels correlating significantly with poor gemcitabine therapeutic response. Mechanistically, we unveiled that H3K18la transcriptionally activates HNRNPC expression. Both in vitro and in vivo functional studies confirmed that HNRNPC silencing substantially restores gemcitabine sensitivity in gemcitabine‐resistant PDAC. Functioning as an m6A “reader”, HNRNPC stabilizes TNF receptor‐associated factor 6 (TRAF6) mRNA through recognizing m6A modification, consequently leading to TRAF6‐mediated autophagy activation and gemcitabine resistance in PDAC. Furthermore, HNRNPC concurrently stabilizes aldehyde dehydrogenase 1 family member A3 (ALDH1A3) mRNA via similar m6A‐dependent mechanisms, driving metabolic reprogramming through glycolytic pathway activation. This metabolic shift generates a positive feedback loop by increasing lactate production and subsequent histone lactylation, thereby amplifying HNRNPC expression. Notably, therapeutic inhibition of ALDH1A3 synergistically suppressed PDAC proliferation when combined with gemcitabine in PDX, highlighting the potential for targeting therapeutic strategies.

## 2 Results

### 2.1 HNRNPC is Upregulated in Gemcitabine‐Resistant PDAC and Correlates with Poor Gemcitabine Therapy Response

**Figure 1:**
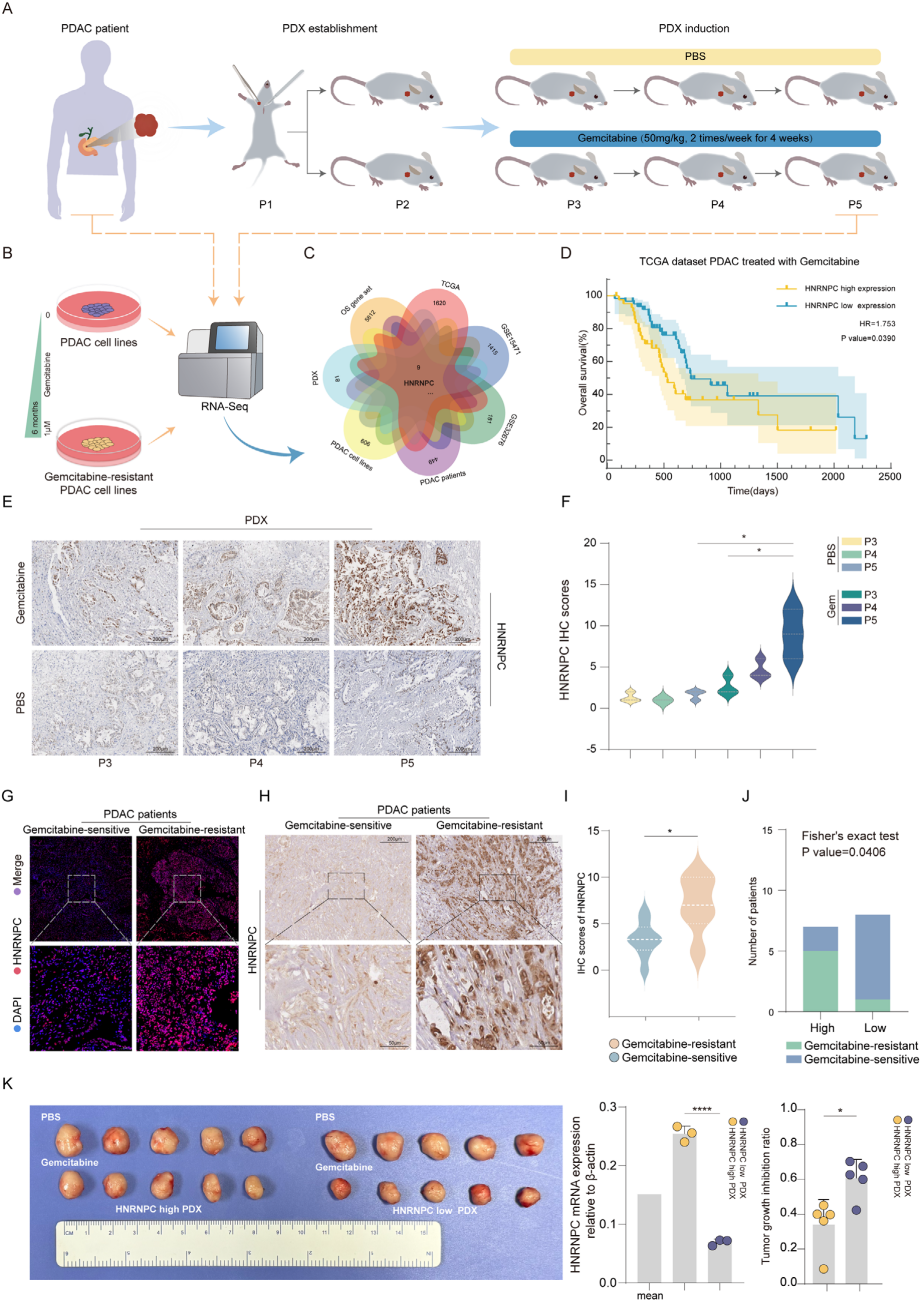
HNRNPC is upregulated in gemcitabine‐resistant PDAC and correlates with poor gemcitabine therapy response. A) Schematic diagram of the gemcitabine‐resistant (GR) PDAC PDX models establishment and the RNA sequencing of PDAC patients and PDX. B) Schematic diagram of the induction and RNA sequencing of gemcitabine‐resistant PDAC cell lines. C) A Venn diagram intersecting with multiple datasets demonstrated the potential genes related to gemcitabine‐resistant PDAC. D) Kaplan–Meier survival curves revealed the correlation between HNRNPC expression and overall survival of gemcitabine‐treated PDAC patients from the TCGA dataset. E) Representative images of IHC staining for HNRNPC in different passages of PDX that underwent PBS or gemcitabine. IHC scores were calculated by multiplying the staining intensity grade (negative, 0; mild, 1; moderate, 2; severe, 3) by the percentage of positively stained tumor area (≤ 25%, 1; >25% and ≤ 50%, 2; >50% and ≤ 75%, 3; >75%, 4). F) The statistical analysis of IHC staining for HNRNPC scores in different passages of PDX that underwent PBS or gemcitabine. ^*^ *p* < 0.05 according to Student's *t*‐test. G) Representative images of IF staining for HNRNPC in gemcitabine‐sensitive or resistant PDAC patients. IF scores were measured by fluorescence intensity with ImageJ. H) Representative images of IHC staining for HNRNPC in gemcitabine‐sensitive or resistant PDAC patients. IHC scores were calculated by multiplying the staining intensity grade (negative, 0; mild, 1; moderate, 2; severe, 3) by the percentage of positively stained tumor area (≤ 25%, 1; > 25% and ≤ 50%, 2; >50% and ≤ 75%, 3; >75%, 4). I) The statistical analysis of IHC staining for HNRNPC scores in gemcitabine‐sensitive or resistant PDAC patients is shown. ^*^ *p* < 0.05 according to Student's *t*‐test. J) The correlation between HNRNPC expression and response to gemcitabine was analyzed using Fisher's exact test. K) The expression of HNRNPC in two PDX models was detected by qPCR, and the tumor images of each corresponding group under the treatment of PBS (sample size, n = 5) or gemcitabine (sample size, n = 5), with the tumor growth inhibition ratio displayed. ^*^ *p* < 0.05; ^****^ *p* < 0.0001 according to Student's *t*‐test. The data are shown as mean ± SD.

To investigate the molecular mechanisms underlying gemcitabine resistance in PDAC, we established gemcitabine‐resistant cell lines, BxPC‐3(GR)/CFPAC‐1(GR), and patient‐derived xenograft (PDX) models (**Figure** **1**A,B; Figure S1A,D, Supporting Information). Comprehensive genomic sequencing of pancreatic tumor/normal tissues, gemcitabine‐resistant/wild‐type cell lines, and gemcitabine‐resistant/sensitive PDX models, integrated with bioinformatics analysis of TCGA, GEO databases, and survival‐associated gene sets of PDAC, revealed significant upregulation of HNRNPC in gemcitabine‐resistant tumors, correlating with worse overall survival in patients treated with gemcitabine (Figure 1C,D). Western blot analysis confirmed elevated HNRNPC expression in gemcitabine‐resistant cells (Figure S1B, Supporting Information). Notably, stepwise gemcitabine induction demonstrated a dose‐dependent increase in HNRNPC protein levels (Figure S1C, Supporting Information). Next, immunohistochemistry (IHC) demonstrated progressive HNRNPC upregulation during gemcitabine‐induced PDX chemoresistance development (P3‐P5) (Figure 1E,F).

Clinical validation through IHC and immunofluorescence (IF) of patient specimens showed marked HNRNPC overexpression in gemcitabine‐resistant vs sensitive tumors. Then, patients were divided into high and low HNRNPC expression groups according to the medium score, and the results showed a 5:2 (gemcitabine resistant: sensitive) ratio in the HNRNPC‐high group vs a 1:7 (gemcitabine resistant: sensitive) ratio in the HNRNPC‐low group (with P = 0.0406 by Fisher's exact test) (Figure 1G–J).

To validate the above findings, we performed quantitative real‐time PCR (qPCR) analysis on the collected PDX tumor tissues and stratified them into high and low HNRNPC expression groups. Subsequently, PDX models were established from each group and treated with gemcitabine. Ultimately, the HNRNPC high‐expression group exhibited significantly greater tolerance to gemcitabine compared to the low‐expression group (Figure 1K). These multi‐level validations across cellular models, clinical samples, and PDX systems collectively establish HNRNPC as a critical gene of gemcitabine resistance in PDAC, correlating with poor gemcitabine therapy response.

### 2.2 HNRNPC Expression is Activated by Histone Lactylation

**Figure 2:**
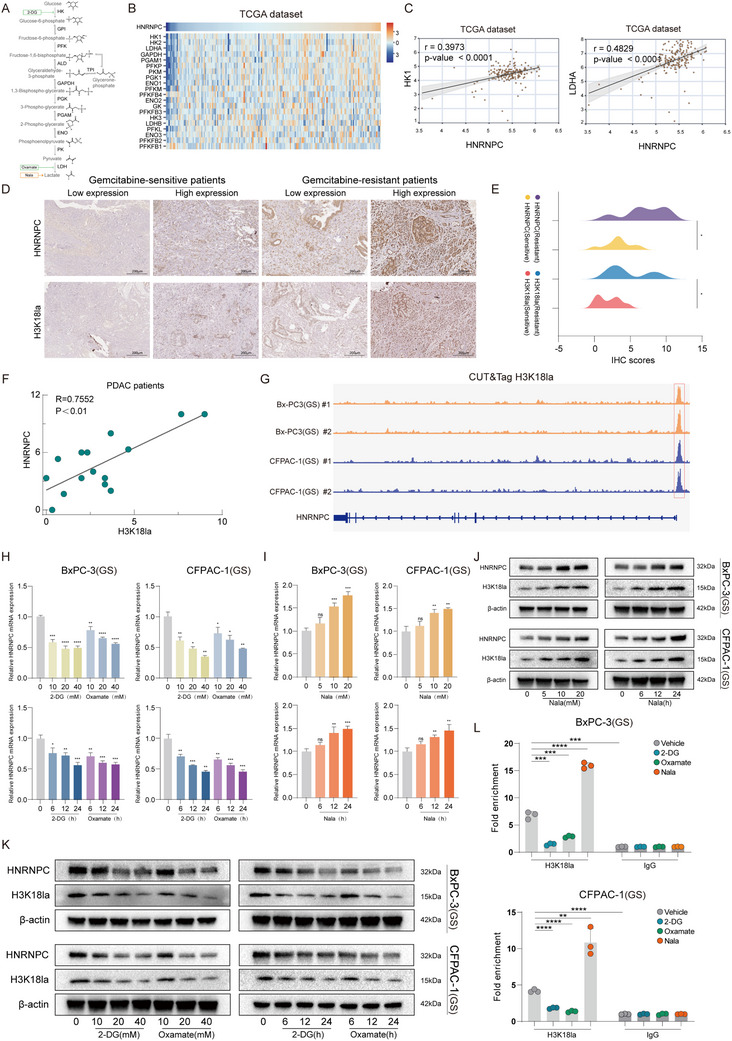
HNRNPC expression is activated by histone lactylation. A) Diagram of the glycolysis pathway and the regulation. B) Heatmap in the TCGA dataset depicted the correlation between HNRNPC and metabolic enzymes of the glycolysis pathway in PDAC patients. C) Scatter plots showed the correlation between HNRNPC and HK1/LDHA, analyzed with the TCGA dataset. D) Representative images of IHC staining for HNRNPC/H3K18la in gemcitabine‐sensitive or resistant PDAC patients. IHC scores were calculated by multiplying the staining intensity grade (negative, 0; mild, 1; moderate, 2; severe, 3) by the percentage of positively stained tumor area (≤ 25%, 1; > 25% and ≤ 50%, 2; >50% and ≤ 75%, 3; >75%, 4). E) IHC scores staining for HNRNPC/H3K18la in gemcitabine‐sensitive or resistant PDAC patients. ^*^ *p* < 0.05 according to Student's *t*‐test. F) Scatter plots showed the correlation between HNRNPC and H3K18la IHC scores. G) H3K18la enrichment peaks on the HNRNPC promoter region in PDAC cells (GS) identified by CUT&Tag. H) HNRNPC mRNA expression level evaluated with qPCR under 2DG/Oxamate treatment in PDAC cells (GS). ^*^ *p* < 0.05; ^**^ *p* < 0.01; ^***^ *p* < 0.001; ^****^ *p* < 0.0001 according to Student's *t*‐test. I) HNRNPC mRNA expression level evaluated with qPCR under Nala treatment in PDAC cells (GS). ^*^ *p* < 0.05; ^**^ *p* < 0.01; ^***^ *p* < 0.001; ^****^ *p* < 0.0001 according to Student's *t*‐test. J) HNRNPC and H3K18la levels identified with western blot subjected to Nala. K) HNRNPC and H3K18la levels identified with western blot subjected to 2DG/Oxamate. L) ChIP‐qPCR analysis of H3K18la enrichment on the HNRNPC promoter region applied with vehicle/2‐DG/Oxamate/Nala in PDAC cells (GS). ^*^ *p* < 0.05; ^**^ *p* < 0.01; ^***^ *p* < 0.001; ^****^ *p* < 0.0001 according to Student's *t*‐test. The data are shown as mean ± SD. Abbreviation: GS, gemcitabine‐sensitive; GR, gemcitabine‐resistant.

Increased glycolysis, a hallmark of tumor metabolic reprogramming, facilitates lactate accumulation (**Figure** **2**A).^[^ ^18^, ^19^ ^]^ Emerging evidence suggests that lactylation functions as a novel histone post‐translational modification regulating gene transcription, with histone lactylation playing critical roles in malignant tumors.^[^ ^14^, ^20^ ^]^ Among those histone lactylation that mainly occur at lysine in H2A, H2B, H3, and H4, H3K18la was identified as the most common histone lactylation site.^[^ ^15^ ^]^ To investigate the mechanism underlying HNRNPC overexpression in gemcitabine‐resistant PDAC, we first analyzed TCGA data and identified significant positive correlations between HNRNPC expression and key glycolytic enzymes (Figure 2B,C; Figure S2A–C, Supporting Information). Subsequently, IHC analysis of patient tissues revealed a positive association between HNRNPC expression levels and H3K18la (Figure 2D–F). CUT&Tag using anti‐H3K18la antibody in gemcitabine‐sensitive BxPC‐3(GS) and CFPAC‐1(GS) cells demonstrated prominent enrichment peaks at the HNRNPC promoter region (Figure 2G). To validate these findings, we treated PDAC cells (GS) with glycolytic inhibitors, 2‐deoxy‐D‐glucose (2‐DG)/Oxamate, and lactate stimulator Nala. qPCR revealed dose‐ and time‐dependent decreases in HNRNPC mRNA levels following lactate inhibition (Figure 2H), while Nala treatment induced upregulation of HNRNPC mRNA levels (Figure 2I). Western blot analysis confirmed corresponding changes in HNRNPC protein expression (Figure 2J,K). Then we conducted a dual‐luciferase reporter gene assay to directly verify the function of the HNRNPC promoter region. Based on the specific region of the HNRNPC promoter rich in H3K18la modification shown by CUT&Tag, we designed a dual‐luciferase reporter vector (Figure S2D, Supporting Information) and transfected this reporter vector into pancreatic cancer cells. The results showed that when lactate was presented, the promoter sequence in this enrichment peak region exhibited a significant enhancement of luciferase activity (Figure S2E, Supporting Information). It indicates that these peaks are located in the functional regulation zone. Moreover, chromatin immunoprecipitation quantitative PCR (ChIP‐qPCR) confirmed reduced H3K18la enrichment at the HNRNPC promoter under glycolytic inhibition, whereas Nala stimulation enhanced H3K18la enrichment (Figure 2L). These results indicate that histone lactylation transcriptionally activates HNRNPC in PDAC.

### 2.3 HNRNPC Knockdown Enhances Gemcitabine Efficacy Against PDAC In Vitro and In Vivo

**Figure 3:**
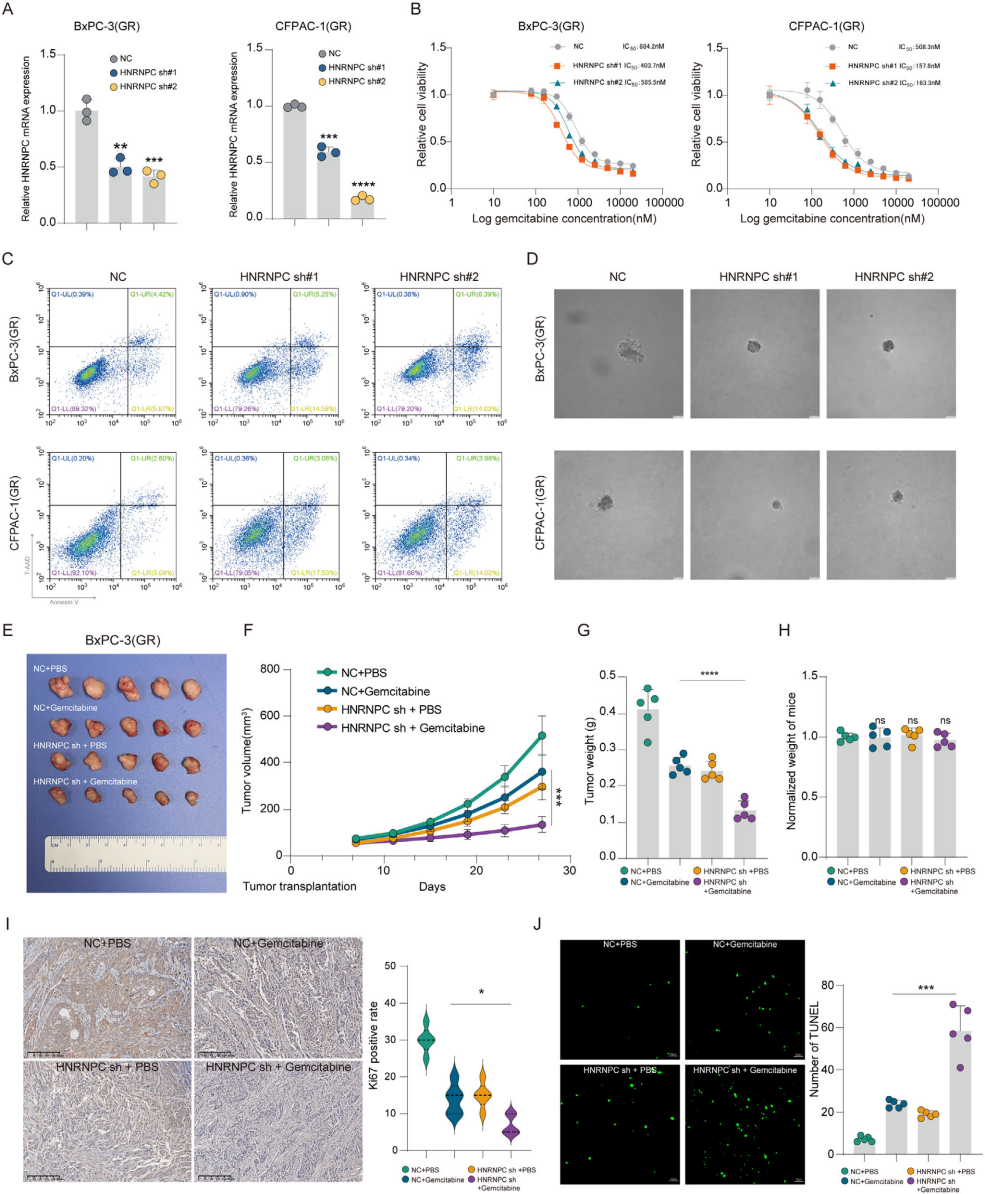
HNRNPC knockdown enhances gemcitabine efficacy against PDAC in vitro and in vivo. A) HNRNPC expression after HNRNPC knockdown in PDAC cells detected by qPCR. ^*^ *p* < 0.05; ^**^ *p* < 0.01; ^***^ *p* < 0.001; ^****^ *p* < 0.0001 according to Student's *t*‐test. B) The IC50 value following HNRNPC knockdown in PDAC cells. C) Apoptosis assay in PDAC cells treated with 1 µM gemcitabine. D) Tumor spheres formation of PDAC cells under the treatment of 1 µM gemcitabine. E) CDX tumors from BxPC‐3(GR) (sample size, n = 5 each group). F) Tumor growth curves of each group. ^***^ *p* < 0.001 according to Student's t‐test. G) Tumor weight of each group. ^****^ *p* < 0.0001 according to Student's *t*‐test. H) The final weight of mice in each group was normalized to the control group. ns (no significance) according to Student's *t*‐test. I) Representative images of IHC staining for Ki67 and statistical analysis in tumors from each group. ^***^ *p* < 0.001 according to Student's *t*‐test. J) Representative images of TUNEL and statistical analysis in tumors with different treatments. ^*^ *p* < 0.05 according to Student's *t*‐test. The data are shown as mean ± SD.

To investigate the functional role of HNRNPC in gemcitabine resistance of PDAC, we established stable HNRNPC‐knockdown cell lines using shRNA in BxPC‐3(GR) and CFPAC‐1(GR) (**Figure** **3**A; Figure S3A, Supporting Information). Cell viability assays revealed that HNRNPC knockdown significantly reduced the half‐maximal inhibitory concentration (IC50) of gemcitabine in PDAC cells (Figure 3B). Subsequent flow cytometric analysis demonstrated a significant increase in apoptotic rates in HNRNPC‐knockdown cells following 48 h gemcitabine treatment (Figure 3C; Figure S3C, Supporting Information). Similarly, EdU assays showed markedly suppressed proliferation activity in HNRNPC‐knockdown cells under gemcitabine treatment (Figure S3B, Supporting Information). Consistent with these findings, soft agar colony formation assays indicated that HNRNPC‐knockdown cells generated substantially smaller tumor spheroids when exposed to the same gemcitabine concentration (Figure 3D; Figure S3D, Supporting Information). Then we conducted parallel functional experiments in the parental gemcitabine‐sensitive pancreatic cancer cells. IC50 and Edu assay show that knockdown of HNRNPC in these sensitive parental cells similarly resulted in a significant enhancement of gemcitabine sensitivity, whereas its overexpression conversely reduced drug sensitivity (Figure S3E,F, Supporting Information).

To evaluate the effect of HNRNPC expression in gemcitabine‐resistant PDAC in vivo, we subcutaneously injected BALB/c‐nude mice with either HNRNPC‐knockdown or wild‐type gemcitabine‐resistant PDAC cells to establish cell line‐derived xenograft (CDX) models. Comparative analysis revealed that HNRNPC‐knockdown CDX tumors displayed enhanced gemcitabine sensitivity. Specifically, the combination of HNRNPC knockdown and gemcitabine treatment resulted in significantly reduced tumor volume and tumor weight relative to the gemcitabine monotherapy group. (Figure 3E–H). Consistent with these phenotypic observations, IHC analysis demonstrated decreased Ki67‐positive rate in HNRNPC‐knockdown CDX tumors treated with gemcitabine, indicative of suppressed proliferation (Figure 3I). Concurrently, TdT‐mediated dUTP Nick‐End Labeling (TUNEL) assays showed increased apoptotic cell numbers in the HNRNPC‐knockdown with gemcitabine treatment group (Figure 3J). These findings suggest that HNRNPC downregulation restores gemcitabine sensitivity in PDAC.

### 2.4 HNRNPC Activates Autophagy by Stabilizing TRAF6 mRNA Through m6A‐Dependent Mechanisms in PDAC

**Figure 4:**
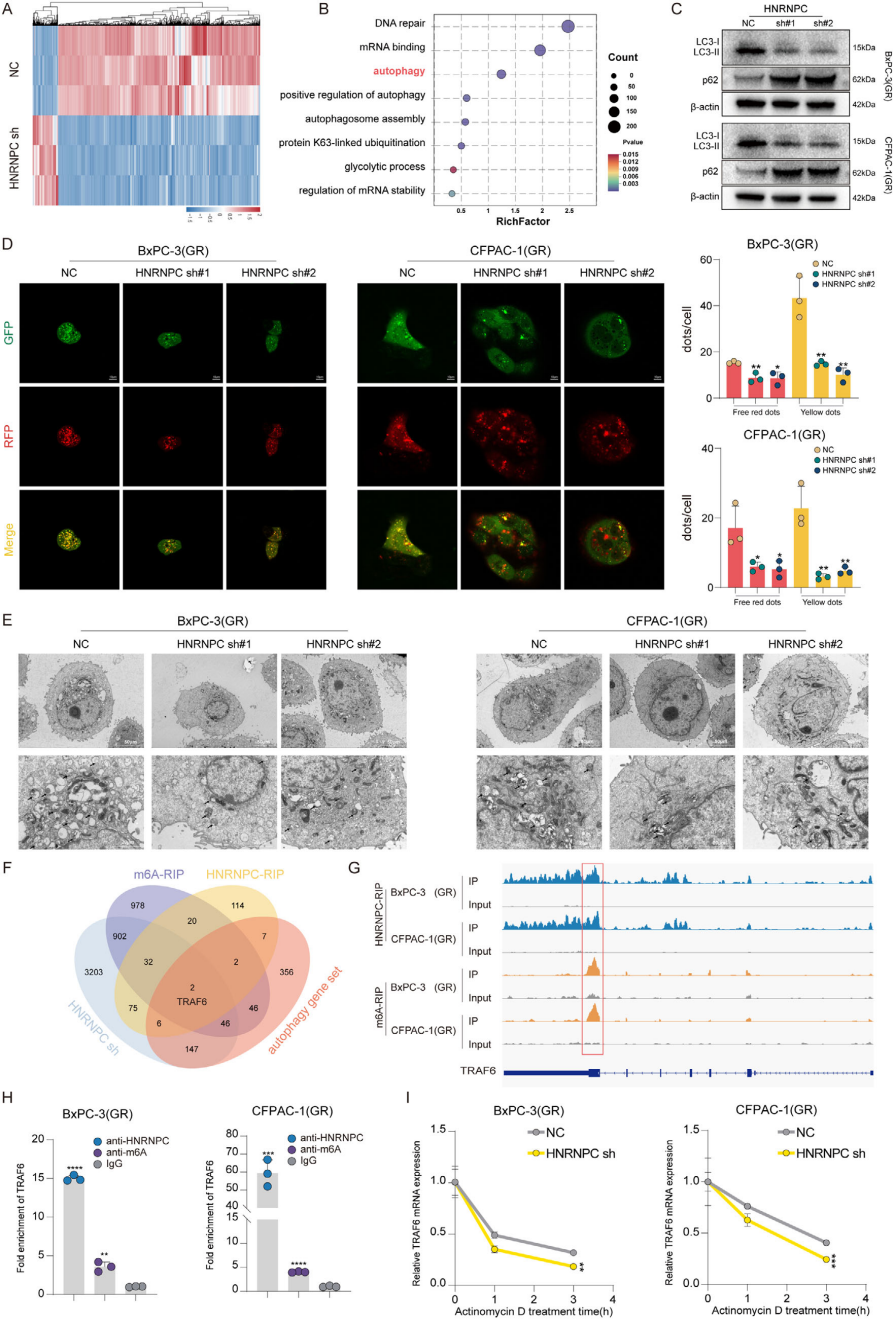
HNRNPC activates autophagy by stabilizing TRAF6 mRNA through m6A‐dependent mechanisms in PDAC. A) Heatmap of PDAC cells RNA sequencing. B) GO enrichment analysis of PDAC cells RNA sequencing differential expression genes. C) Western blot assessed the autophagy markers level in PDAC cells treated with gemcitabine at 1 µM. D) Representative images of autophagy formation (shown as red/yellow dots) documented by mRFP‐GFP‐LC3 puncta assay and statistical analysis in PDAC cells applied with 1 µM gemcitabine. ^*^ *p* < 0.05; ^**^ *p* < 0.01; ^***^ *p* < 0.001; ^****^ *p* < 0.0001 according to Student's *t*‐test. E) Representative transmission electron microscopy (TEM) images of autophagy level (indicated with arrows) in PDAC cells in the presence of 1 µM gemcitabine. F) A Venn diagram intersecting with multiple datasets implied TRAF6 as the target of HNRNPC to regulate autophagy in PDAC. G) RIP‐sequencing discovered the m6A modification and HNRNPC binding on TRAF6 mRNA. H) RIP‐qPCR detected TRAF6 mRNA m6A modification and HNRNPC binding. ^*^ *p* < 0.05; ^**^ *p* < 0.01; ^***^ *p* < 0.001; ^****^ *p* < 0.0001 according to Student's *t*‐test. I) qPCR analysis of TRAF6 mRNA levels after actinomycin D treatment in PDAC cells. ^**^ *p* < 0.01; ^***^ *p* < 0.001 according to Student's *t*‐test. The data are shown as mean ± SD.

To investigate the mechanistic basis of HNRNPC‐mediated gemcitabine resistance in PDAC, HNRNPC‐knockdown cell lines were subjected to whole‐transcriptome RNA sequencing (**Figure** **4**A). Subsequent bioinformatic interrogation of differentially expressed genes through Gene Ontology (GO) and Kyoto Encyclopedia of Genes and Genomes (KEGG) pathway analyses demonstrated significant enrichment of autophagy‐related pathways (Figure 4B; Figure S4A, Supporting Information). This molecular signature suggests a potential regulatory role of HNRNPC in conferring gemcitabine resistance through autophagy modulation in PDAC. While autophagy represents an evolutionarily conserved catabolic mechanism essential for pathogen eradication and cellular homeostasis through lysosomal degradation, mounting evidence demonstrates its pivotal role in promoting therapeutic resistance across diverse malignancies.^[^ ^21^ ^]^ To validate this hypothesis, western blot analysis revealed a significant reduction in LC3‐II and increased p62 protein levels, certified autophagy markers, in HNRNPC‐knockdown BxPC‐3(GR) and CFPAC‐1(GR) (Figure 4C). This finding was corroborated by the mRFP‐GFP‐LC3 puncta assay, demonstrating substantial inhibition of autophagic flux following HNRNPC knockdown (Figure 4D). Ultrastructural confirmation was obtained through transmission electron microscopy (TEM), which visually documented diminished autophagy formation in HNRNPC knockdown gemcitabine‐resistant PDAC cells (Figure 4E).

HNRNPC regulates mRNA metabolism by recognizing m6A methylation modification on target transcripts, stabilizing mRNA through binding interaction.^[^ ^12^ ^]^ To elucidate the mechanistic framework through which HNRNPC modulates autophagic activity in gemcitabine‐resistant PDAC, we performed RNA immunoprecipitation sequencing (RIP‐Seq) using anti‐HNRNPC and anti‐m6A antibodies. Integrated analysis of HNRNPC knockdown RNA sequencing profiles with autophagy‐related gene sets, coupled with Venn diagram intersection of RIP‐Seq, identified TNF receptor‐associated factor 6 (TRAF6) as a candidate mediator of HNRNPC‐regulated autophagy in gemcitabine‐resistant PDAC (Figure 4F). TRAF6 has been molecularly characterized not only as a critical regulator of autophagic pathways but also as a potent contributor to tumor malignancy, as evidenced by prior oncological studies.^[^ ^22^, ^23^, ^24^ ^]^ Building upon these findings, we conducted systematic validation to determine whether HNRNPC confers gemcitabine resistance in PDAC through TRAF6‐mediated autophagy regulation. qPCR analysis of HNRNPC‐knockdown cell lines demonstrated significant downregulation of TRAF6 mRNA expression, a finding corroborated at the protein level through western blot validation (Figure S4B,C, Supporting Information). Mechanistically, computational interrogation of HNRNPC‐RIP and m6A‐RIP sequencing datasets revealed binding of TRAF6 mRNA (Figure 4G). These suggest a molecular mechanism that HNRNPC recognizes and binds to TRAF6 mRNA, thereby enhancing mRNA stability and consequently upregulating TRAF6 expression. To substantiate this mechanistic hypothesis, we performed RNA immunoprecipitation quantitative PCR (RIP‐qPCR) in gemcitabine‐resistant PDAC cell lines using anti‐m6A and anti‐HNRNPC antibodies. This analysis confirmed significant enrichment of m6A modification and HNRNPC binding on TRAF6 mRNA (Figure 4H). Subsequently, actinomycin D‐mediated mRNA decay assays demonstrated markedly reduced TRAF6 mRNA stability when HNRNPC was knocked down in PDAC cell lines (Figure 4I). To further clarify that HNRNPC regulates the m6A modification site of TRAF6 mRNA, we identified the m6A modification site on TRAF6 mRNA in pancreatic cancer cells by m6A RIP‐Seq and constructed the corresponding dual‐luciferase plasmid (Figure S4D, Supporting Information). The results showed that when HNRNPC was knocked down in the wild‐type (WT), the luciferase activity was significantly reduced. However, in the m6A modification site mutant type (the classic m6A motif RRACH is mutated to RRGCH. R = G/A, H = A/C/U), knocking down the expression of HNRNPC, luciferase activity did not show significant changes (Figure S4E, Supporting Information). These findings collectively establish a regulatory axis wherein HNRNPC recognizes and binds to m6A modification on TRAF6 mRNA, thereby enhancing mRNA stability to upregulate its expression. This molecular interaction ultimately potentiates autophagic activity in PDAC, providing a mechanistic foundation for HNRNPC‐mediated gemcitabine resistance.

### 2.5 TRAF6 is Responsible for HNRNPC‐Mediated Gemcitabine Resistance in PDAC

**Figure 5:**
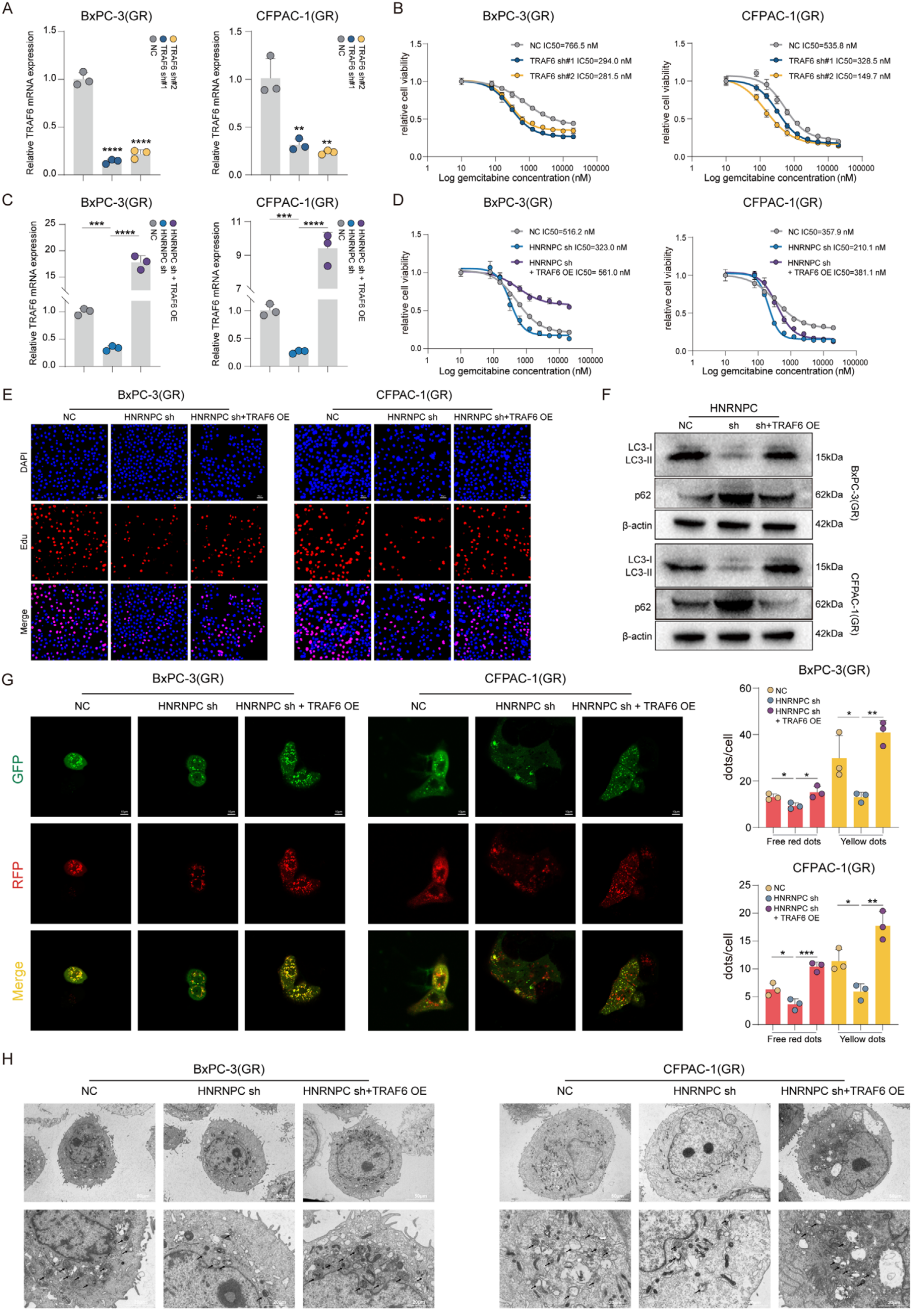
TRAF6 is responsible for HNRNPC‐mediated gemcitabine resistance in PDAC. A) TRAF6 expression after TRAF6 knockdown in PDAC cells was verified by qPCR. ^*^ *p* < 0.05; ^**^ *p* < 0.01; ^***^ *p* < 0.001; ^****^ *p* < 0.0001 according to Student's *t*‐test. B) The IC50 value following TRAF6 knockdown in PDAC cells. C) TRAF6 mRNA level confirmed by qPCR in PDAC cells, as TRAF6 is overexpressed. ^***^ *p* < 0.001; ^****^ *p* < 0.0001 according to Student's *t*‐test. D) The IC50 value along with TRAF6 overexpression in PDAC cells. E) Representative images of the Edu assay evaluating proliferation in PDAC cells in the presence of 1 µM gemcitabine. F) Western blot assessed autophagy marker levels in PDAC cells treated with gemcitabine at 1 µM. G) Representative images of autophagy formation (shown as red/yellow dots) monitored by mRFP‐GFP‐LC3 puncta assay along with statistical analysis in PDAC cells applied with 1 µM gemcitabine. ^*^ *p* < 0.05; ^**^ *p* < 0.01; ^***^ *p* < 0.001; ^****^ *p* < 0.0001 according to Student's *t*‐test. H) Representative transmission electron microscopy (TEM) images of autophagy level (indicated with arrows) in PDAC cells treated with 1 µM gemcitabine. The data are shown as mean ± SD.

To elucidate TRAF6's functional role in gemcitabine resistance mechanisms in PDAC, we established stable TRAF6 knockdown in BxPC‐3(GR)/CFPAC‐1(GR) cell lines using shRNA (**Figure** **5**A; Figure S5A, Supporting Information). Subsequent cell viability assays demonstrated a substantial reduction in gemcitabine's IC50 following TRAF6 knockdown (Figure 5B). Consistent with these findings, EdU proliferation assays demonstrated significantly reduced cellular proliferative capacity in TRAF6 knockdown cells following 48 h of gemcitabine treatment (Figure S5B, Supporting Information). To validate whether HNRNPC promotes gemcitabine resistance in PDAC via TRAF6, we conducted rescue experiments by overexpressing TRAF6 in HNRNPC knockdown BxPC‐3(GR) /CFPAC‐1(GR) cell lines (Figure 5C; Figure S5C, Supporting Information). Cell viability profiling revealed that HNRNPC knockdown substantially reduced gemcitabine IC50 values in PDAC cell lines, while TRAF6 overexpression in this genetic background reversed this gemcitabine sensitivity effect, restoring IC50 levels (Figure 5D). Parallel EdU assays corroborated this functional rescue; gemcitabine‐treated HNRNPC‐knockdown cells exhibited suppressed proliferation, whereas concurrent TRAF6 overexpression in these cells effectively restored proliferation (Figure 5E; Figure S5D, Supporting Information). Consistent with these observations, western blot analysis of LC3 and p62 protein demonstrated that TRAF6 overexpression effectively rescued the autophagy suppression induced by HNRNPC knockdown (Figure 5F). Meanwhile, the mRFP‐GFP‐LC3 puncta assay showed that TRAF6 overexpression can activate the autophagic flux. Complementary validation confirmed the restoration of autophagic flux following the overexpression of TRAF6 in HNRNPC knockdown PDAC cells (Figure 5G, Figure S5H,I, Supporting Information). Ultrastructural analysis via TEM further revealed significant recovery of autophagy formation in HNRNPC‐knockdown cells with TRAF6 overexpression (Figure 5H). Relevant literature reports that TRAF6 can catalyze the ubiquitination of the autophagy‐related protein Beclin1, thereby blocking the interaction between Beclin1 and Bcl2, and promoting the autophagy response.^[^ ^22^, ^25^, ^26^ ^]^ We used TRAF6 antibody for co‐immunoprecipitation and found that TRAF6 interacted with Beclin1 (Figure S5E, Supporting Information). Subsequently, western blot detection was performed on the ubiquitination modification of Beclin1, and it was found that the ubiquitination modification of Beclin1 decreased with the knockdown of TRAF6 (Figure S5F, Supporting Information), and at the same time, with the knockdown of TRAF6, we found that the protein interaction between Bcl2 and Beclin1 was enhanced (Figure S5G, Supporting Information). The results indicate that TRAF6 mediates gemcitabine resistance in pancreatic cancer by promoting the ubiquitination modification of Beclin1, thereby blocking interaction between Beclin‐1 and Bcl2. This multilevel experimental evidence establishes that HNRNPC confers gemcitabine resistance in PDAC through TRAF6 regulation in autophagy potentiation.

### 2.6 HNRNPC Maintains ALDH1A3 mRNA Stability Reinforcing H3K18la to Establish a Positive Feedback Loop

**Figure 6:**
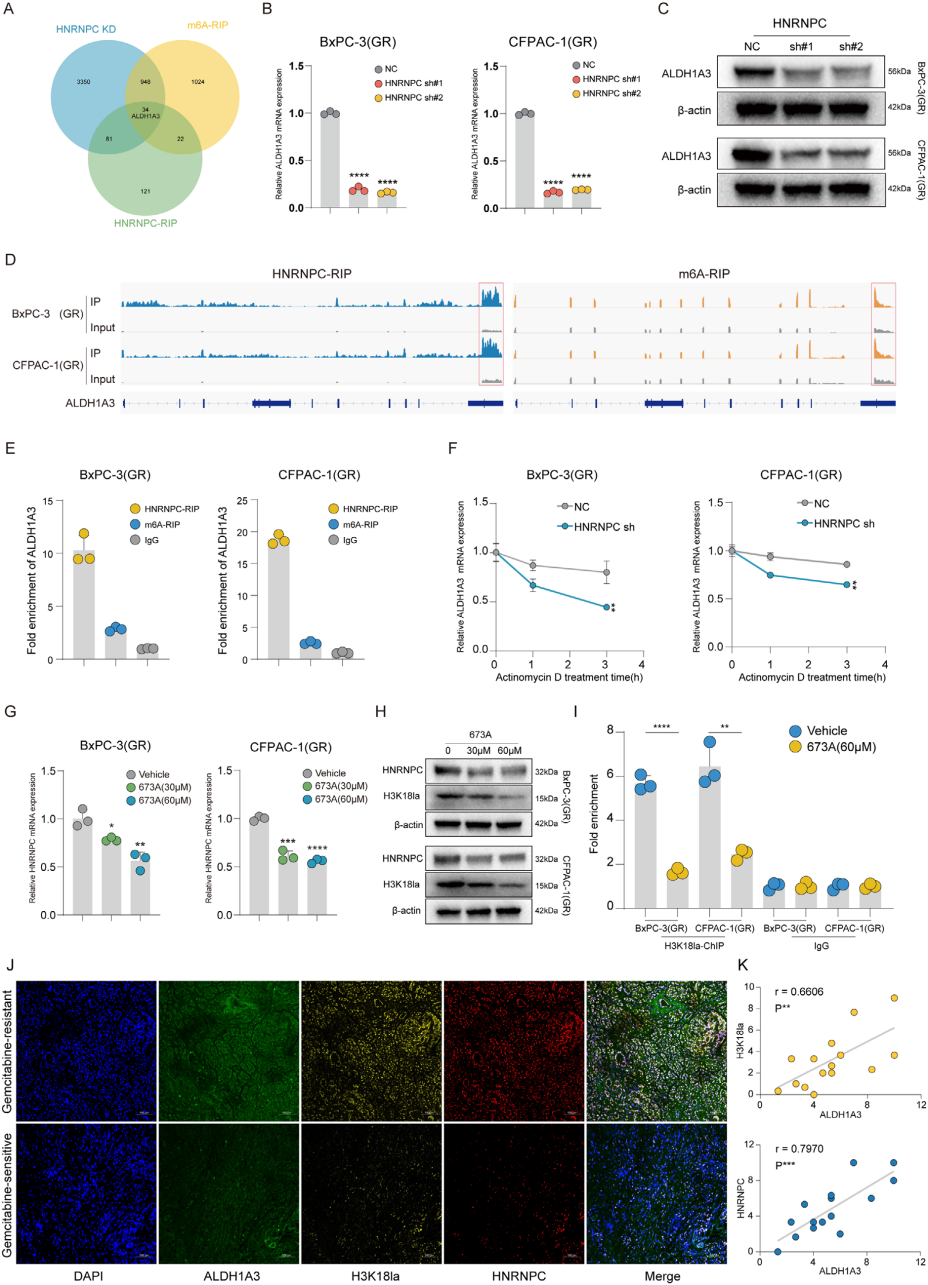
HNRNPC maintains ALDH1A3 mRNA stability, reinforcing H3K18la to establish a positive feedback loop. A) Venn diagram intersection of HNRNPC knockdown (KD), m6A‐RIP, and HNRNPC‐RIP sequencing data indicated ALDH1A3 as the target of HNRNPC. B) qPCR quantified ALDH1A3 mRNA expression in HNRNPC knockdown PDAC cells. ^*^ *p* < 0.05; ^**^ *p* < 0.01; ^***^ *p* < 0.001; ^****^ *p* < 0.0001 according to Student's *t*‐test. C) Western blot confirmed ALDH1A3 expression in HNRNPC‐knockdown. D) RIP‐sequencing revealed the m6A modification and HNRNPC binding on ALDH1A3 mRNA. E) RIP‐qPCR detected ALDH1A3 mRNA m6A modification and HNRNPC binding. ^*^ *p* < 0.05; ^**^ *p* < 0.01; ^***^ *p* < 0.001; ^****^ *p* < 0.0001 according to Student's *t*‐test. F) qPCR analysis of ALDH1A3 mRNA level after actinomycin D treatment in PDAC cells. ^*^ *p* < 0.05; ^**^ *p* < 0.01; ^***^ *p* < 0.001; ^****^ *p* < 0.0001 according to Student's *t*‐test. G) HNRNPC expression level quantified with qPCR in PDAC cells after treatment with 673A. ^*^ *p* < 0.05; ^**^ *p* < 0.01; ^***^ *p* < 0.001; ^****^ *p* < 0.0001 according to Student's *t*‐test. H) HNRNPC expression and H3K18la level confirmed by western blot in PDAC cells subjected to 673A. I) ChIP‐qPCR analysis of H3K18la enrichment on the HNRNPC promoter region applied with vehicle or 673A in PDAC cells. ^*^ *p* < 0.05; ^**^ *p* < 0.01; ^***^ *p* < 0.001; ^****^ *p* < 0.0001 according to Student's *t*‐test. J) Representative images of mIF staining for ALDH1A3, H3K18la, and HNRNPC in gemcitabine‐sensitive or resistant PDAC patients. IF scores were measured by fluorescence intensity with ImageJ. K) Correlation analysis between ALDH1A3 and H3K18la/HNRNPC in PDAC patients by IHC. The data are shown as mean ± SD.

Intriguingly, during investigations into the mechanisms underlying HNRNPC‐regulated gemcitabine resistance in PDAC, integrated analysis of HNRNPC‐knockdown sequencing, HNRNPC‐RIP sequencing, and m6A‐RIP sequencing data revealed that aldehyde dehydrogenase 1 family member A3 (ALDH1A3), which has been reported to exert a function in upregulation of glycolysis,^[^ ^27^ ^]^ emerges as a potential downstream target in HNRNPC‐regulated gemcitabine resistance in PDAC (**Figure** **6**A). To validate this hypothesis, we assessed ALDH1A3 expression levels by qPCR and western blot in HNRNPC‐knockdown BxPC‐3(GR)/CFPAC‐1(GR) cell lines (Figure 3A; Figure S3A, Supporting Information), and demonstrated that HNRNPC knockdown results in significant downregulation of ALDH1A3 expression in gemcitabine‐resistant PDAC (Figure 6B,C). Concurrent analysis of m6A‐RIP and HNRNPC‐RIP sequencing identified m6A modification peaks and HNRNPC binding peaks on ALDH1A3 mRNA (Figure 6D). Subsequent RIP‐qPCR validation using anti‐m6A and anti‐HNRNPC antibodies confirmed the substantial enrichment of m6A modifications and HNRNPC binding on ALDH1A3 mRNA (Figure 6E). Subsequently, we conducted mRNA decay assays using actinomycin D in gemcitabine‐resistant PDAC cell lines, which demonstrated that HNRNPC regulates ALDH1A3 mRNA stability, as evidenced by significantly reduced ALDH1A3 mRNA stability following HNRNPC knockdown (Figure 6F). Further analysis with the TCGA dataset, we confirmed a positive expression correlation between ALDH1A3 and HNRNPC in PDAC patients (Figure S6A, Supporting Information). These findings suggest that HNRNPC can upregulate the expression of the glycolytic enzyme ALDH1A3.

Notably, as we mentioned before, we demonstrated that H3K18la promotes HNRNPC expression. We therefore hypothesize that HNRNPC may activate glycolysis by upregulating ALDH1A3 expression, thereby increasing H3K18la levels and forming a positive feedback loop to further enhance its own expression. To further explore the effect of HNRNPC on cellular energy through ALDH1A3‐mediated metabolic reprogramming, Extracellular Acidification Rate (ECAR) detection revealed that the glycolytic pathway of the cells was inhibited after HNRNPC knockdown, and overexpression of ALDH1A3 can restore the glycolytic level (Figure S6D, Supporting Information). Western blot detection of H3K18la levels in cells yielded consistent results. After knocking down HNRNPC, the level of H3K18la decreased, and overexpression of ALDH1A3 could restore the level of H3K18la. Knockdown of ALDH1A3 effectively reduced the level of H3K18la. Administering lactate on the basis of knockdown of ALDH1A3 could restore the level of H3K18la (Figure S6E, Supporting Information). Then we treated PDAC cells with 673A, which has been confirmed to effectively inhibit ALDH1A3.^[^ ^28^ ^]^ Subsequent qPCR analysis revealed a decrease in HNRNPC expression. (Figure 6G). Furthermore, western blot revealed concomitant reductions in both HNRNPC protein levels and H3K18la levels following 673A treatment (Figure 6H). Subsequently, we performed ChIP‐qPCR and found that the enrichment level of H3K18la at the HNRNPC promoter region was significantly decreased after 673A treatment (Figure 6I). Through IHC and IF, we observed that HNRNPC, ALDH1A3, and H3K18la were significantly elevated in gemcitabine‐resistant PDAC patients. (Figures 2E and 6J; Figure S6B, Supporting Information). Concurrently, we demonstrated a positive correlation among the levels of HNRNPC, ALDH1A3, and H3K18la (Figures 2F and 6K; Figure S6C, Supporting Information). The above results indicate that HNRNPC upregulates ALDH1A3 expression via an m6A‐dependent mechanism, which subsequently increases H3K18la levels, thereby establishing a positive feedback loop that enhances HNRNPC's own expression.

### 2.7 Pharmaceutical 673A Targets Feedback Signal to Potentiate Gemcitabine Efficacy Against Gemcitabine‐Resistant PDAC

**Figure 7:**
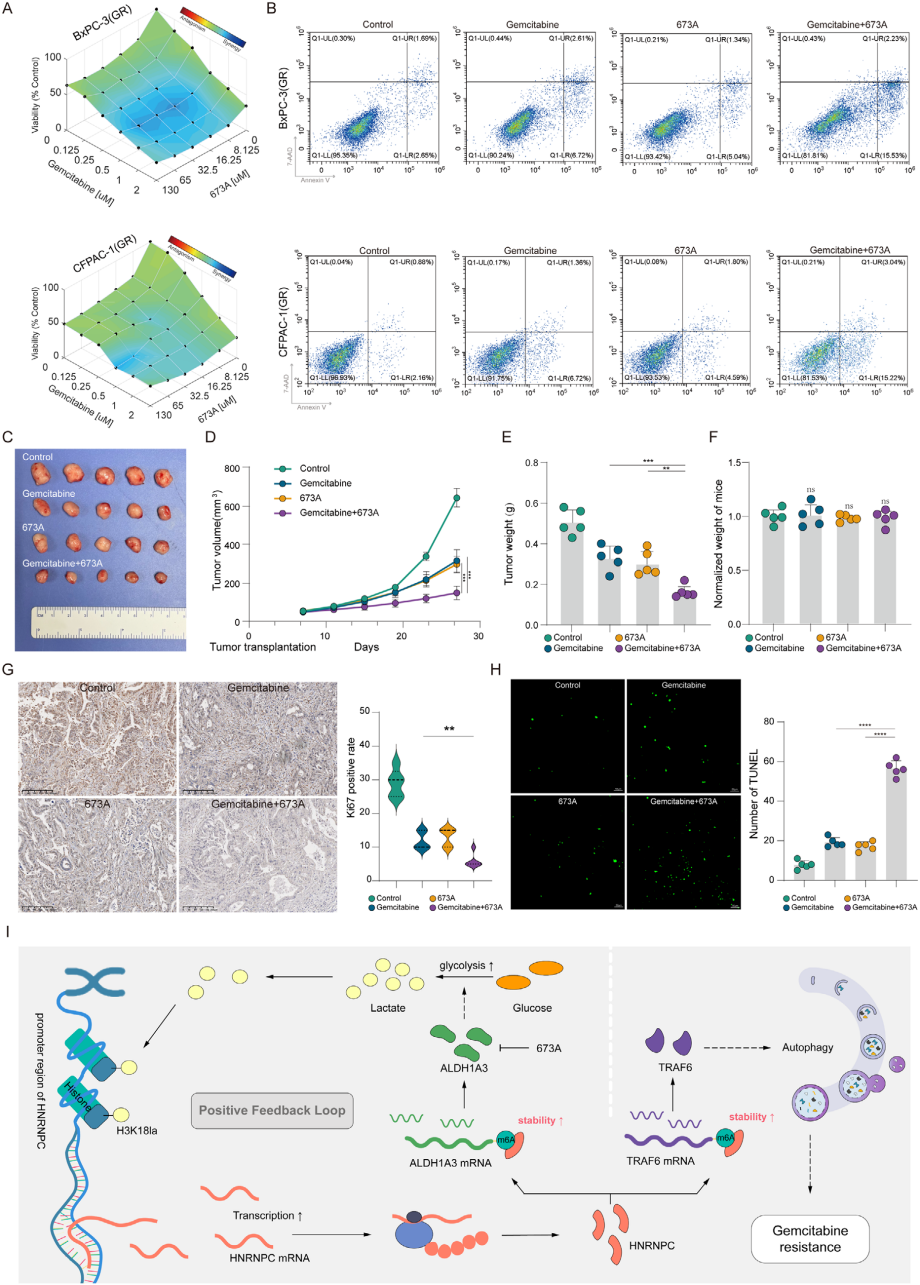
Pharmaceutical 673A targets the feedback signal to potentiate gemcitabine efficacy against gemcitabine‐resistant PDAC. A) Synergy effect validated by the Loewe model in combination of gemcitabine and 673A in PDAC cells. B) Apoptosis assay in PDAC cells administered at gemcitabine (1 µM), 673A (30 µM), or combination. C) PDX models bearing tumor received vehicle, gemcitabine, 673A, or combination, tumors were displayed (sample size, n = 5 each group). D) Tumor growth curves of each group. ^***^ *p* < 0.001 according to Student's *t*‐test. E) Tumor weight of each group. ^**^ *p* < 0.01; ^***^ *p* < 0.001 according to Student's *t*‐test. F) The normalized weight of mice in each group. ^*^ *p* < 0.05; ^**^ *p* < 0.01; ^***^ *p* < 0.001; ^****^ *p* < 0.0001; ns (no significance) according to Student's *t*‐test. G) Representative images of IHC staining for Ki67 and statistical analysis in PDX tumors from each group. ^**^ *p* < 0.01 according to Student's *t*‐test. H) Representative images of TUNEL and statistical analysis in PDX tumors with different treatments. ^***^ *p* < 0.001 according to Student's *t*‐test. I) Schematic representation of the function and potential mechanism of HNRNPC in gemcitabine‐resistant PDAC. The data are shown as mean ± SD. ^*^ *p* < 0.05; ^**^ *p* < 0.01; ^***^ *p* < 0.001 according to Student's *t*‐test.

Building upon these findings, we sought to investigate the clinical translational potential of disrupting the HNRNPC positive feedback loop to reverse gemcitabine resistance in PDAC. Utilizing the ALDH1A3 inhibitor 673A in combination with gemcitabine, we identified synergistic drug interactions in PDAC cell lines through computational analysis with the Loewe additivity model (**Figure** **7**A). Flow cytometric apoptosis assays further confirmed that 673A significantly potentiated gemcitabine‐induced apoptosis in gemcitabine‐resistant PDAC cells (Figure 7B; Figure S7A, Supporting Information). To validate these findings in vivo, we administered 673A, gemcitabine, or their combination to PDX models. Tumor volume measurements obtained every four days demonstrated that the combination therapy of 673A and gemcitabine achieved superior tumor suppression relative to the gemcitabine single‐agent regimen (Figure 7C,D). Consistent with volumetric data, endpoint tumor weight analysis revealed parallel patterns: combination therapy outperformed gemcitabine monotherapy in tumor suppression (Figure 7E). Moreover, no statistically significant intergroup differences in body weight, organ histopathology, and hematology/biochemistry of mice were observed (Figure 7F; Figure S7B,C, Supporting Information). Complementarily, IHC analysis demonstrated substantially lower Ki67 positive rate in the combination therapy group compared to the gemcitabine monotherapy cohort (Figure 7G). Further TUNEL assays revealed a marked increase in apoptosis rates in tumors receiving combination therapy (Figure 7H). Collectively, these findings indicated that 673A exhibits a therapeutic potential effect on reversing gemcitabine resistance in PDAC without significantly increasing side effects.

## 3 Discussion

Cancer has emerged as a leading cause of human morbidity and mortality, with PDAC representing one of the most therapeutically challenging malignancies due to its characteristically high recurrence rates.^[^ ^1^, ^2^ ^]^ Notably, gemcitabine chemoresistance constitutes a predominant driver of this elevated recurrence in PDAC. Thus, there exists an urgent need to elucidate the underlying mechanisms of gemcitabine resistance and develop novel therapeutic strategies to overcome this critical barrier in PDAC treatment.

In this study, we identified HNRNPC as aberrantly overexpressed in gemcitabine‐resistant PDAC, demonstrating a significant correlation with poor gemcitabine therapy response. Both in vitro and in vivo experimental validations confirmed that HNRNPC potentiates gemcitabine resistance in PDAC. Mechanistically, HNRNPC upregulates TRAF6 expression through an m6A modification‐dependent manner, which subsequently activates the intracellular autophagy pathway, thereby conferring the gemcitabine‐resistant phenotype. Concurrently, HNRNPC facilitates intracellular lactate accumulation via m6A‐mediated ALDH1A3 upregulation, subsequently enhancing H3K18la modification. Furthermore, H3K18la transcriptionally activates HNRNPC expression. This process establishes a positive feedback loop that amplifies HNRNPC expression. Based on these findings, we employed the ALDH1A3 inhibitor 673A to disrupt this autoregulatory circuit. Experimental evidence in vitro and in vivo demonstrated that 673A synergistically enhances gemcitabine's cytotoxic effects against PDAC. These findings collectively underscore the translational potential of targeting the HNRNPC‐ALDH1A3 axis as a therapeutic strategy to overcome gemcitabine resistance in PDAC.

As one of the most prevalent RNA modifications, m6A participates in diverse RNA metabolic processes, including stability regulation, splicing, translation, and so on.^[^ ^29^ ^]^ The pivotal m6A “reader”, HNRNPC, regulates mRNA metabolic functions—including stability maintenance and alternative splicing—through its recognition and binding of m6A modification on mRNAs, thereby mediating critical biological processes.^[^ ^11^, ^30^, ^31^ ^]^ Accumulating evidence demonstrates that HNRNPC exerts substantial influence in malignant tumors by modulating mRNA stability via m6A recognition. A study documented that HNRNPC sustains IRAK1 mRNA stability in an m6A‐dependent manner, subsequently activating the mitogen‐activated protein kinase (MAPK) signaling pathway to facilitate glioma progression.^[^ ^12^ ^]^ Concurrently, studies have revealed that HNRNPC promotes epithelial‐mesenchymal transition (EMT) and migration in lung adenocarcinoma (LUAD) by regulating GRB2 mRNA stability through m6A‐dependent mechanisms.^[^ ^11^ ^]^ In our study, we found that HNRNPC is highly expressed in gemcitabine‐resistant PDAC. Both in vivo and in vitro experiments confirmed that HNRNPC promotes gemcitabine resistance in PDAC. Mechanistically, our research demonstrates that HNRNPC exerts its function by recognizing and stabilizing mRNA in an m6A‐dependent manner, thereby upregulating the expression of TRAF6 and ALDH1A3.

Histone lactylation, a novel post‐translational modification discovered in recent years, directly regulates transcriptional activity of target genes, thereby influencing critical biological processes.^[^ ^14^ ^]^ A recent study revealed that colorectal cancer patients resistant to bevacizumab therapy exhibit elevated histone lactylation levels. Significantly, inhibition of histone lactylation enhanced the antitumor effects of bevacizumab.^[^ ^32^ ^]^ Additionally, research demonstrates that cisplatin‐resistant bladder cancer cells exhibit enrichment of H3K18la within the promoter regions of YY1 and YBX1. This lactylation modification transcriptionally activates YBX1 and YBX1 expression, ultimately driving cisplatin resistance in bladder cancer.^[^ ^33^ ^]^ However, the role of histone lactylation in gemcitabine resistance in PDAC remains unclear. In our study, we observed elevated levels of H3K18la in gemcitabine‐resistant PDAC. Furthermore, H3K18la activates the expression of HNRNPC.

Autophagy, a highly conserved catabolic process induced under diverse cellular stress conditions, serves cytoprotective functions by preventing cellular damage and promoting survival during nutrient or energy deprivation, while simultaneously responding to cytotoxic insults.^[^ ^34^ ^]^ Substantial evidence indicates the pivotal involvement of autophagy in tumor chemoresistance. For instance, TAMs/CXCL1 signaling was shown to promote chemoresistance in breast cancer cells through autophagy activation in vitro, whereas CXCL1 silencing enhanced chemosensitivity of paclitaxel‐resistant cells via autophagy inhibition.^[^ ^35^ ^]^ In this study, we confirmed that HNRNPC promotes gemcitabine resistance in PDAC by upregulating autophagy. TRAF6, recognized as a critical regulator of autophagic pathways, has also been implicated in therapeutic resistance mechanisms.^[^ ^22^, ^23^ ^]^ A study demonstrated that in chronic myeloid leukemia, TRAF6 interacts with ULK1 to activate autophagy, thereby driving imatinib resistance through this molecular axis.^[^ ^24^ ^]^ However, the role of TRAF6 in gemcitabine resistance in PDAC remains unclear. In this study, we confirmed that HNRNPC promotes gemcitabine resistance in PDAC by upregulating TRAF6 expression in an m6A‐dependent manner, thereby increasing autophagy.

ALDH1A3 has been documented to mediate tumor malignant phenotypes by activating the glycolytic pathway, thereby augmenting intracellular lactate accumulation. Comparative analyses reveal that mesenchymal glioma stem cells (Mes GSCs) exhibit more aggressive biological behaviors than proneural glioma stem cells (PN GSCs), with significantly elevated glycolytic activity observed in Mes GSCs. Notably, ALDH1A3 is preferentially enriched in Mes GSCs, and its pharmacological inhibition markedly attenuates their proliferative capacity.^[^ ^36^ ^]^ In this study, we discovered that ALDH1A3, as a downstream target of HNRNPC, increases H3K18la levels by modulating glycolysis, thereby forming a positive feedback loop to upregulate HNRNPC expression. The ALDH1A3‐specific inhibitor 673A demonstrates selective efficacy in inducing necroptotic cell death within CD133+ ovarian cancer stem‐like cells (CSCs). Preclinical evaluations confirm that 673A effectively reverses chemotherapy resistance across multiple in vivo ovarian cancer models while significantly synergizing with conventional chemotherapeutic agents.^[^ ^28^ ^]^ Here, we confirmed that compound 673A exhibits a synergistic effect with gemcitabine, and validated in PDX models that 673A significantly enhanced the effect of gemcitabine on PDAC without significantly increasing toxic side effects, highlighting the clinical translational potential of the gemcitabine combination with 673A therapy.

In summary, our study demonstrates that HNRNPC is aberrantly overexpressed in gemcitabine‐resistant PDAC. Mechanistically, HNRNPC recognizes and stabilizes TRAF6 mRNA through m6A modification‐dependent binding, thereby upregulating TRAF6 expression to activate the autophagy pathway and promote gemcitabine resistance. Concurrently, HNRNPC employs an analogous mechanism to enhance ALDH1A3 expression, which activates the glycolytic pathway and elevates intracellular lactate levels. This metabolic reprogramming mediates H3K18 histone lactylation, establishing a positive feedback loop that reinforces HNRNPC self‐expression. Importantly, pharmacological inhibition of ALDH1A3 with compound 673A effectively disrupts this autoregulatory circuit, significantly restoring gemcitabine sensitivity both in vitro and in vivo. Our findings not only elucidate the pivotal role of HNRNPC in driving PDAC gemcitabine resistance but also reveal an intricate crosstalk among m6A epitranscriptomic regulation, autophagy modulation, and histone lactylation‐mediated epigenetic reprogramming. From a therapeutic perspective, this work proposes a novel strategy to overcome gemcitabine resistance by targeting the HNRNPC‐ALDH1A3 regulatory axis, offering promising clinical implications for improving PDAC treatment outcomes.

## 4 Experimental Section

### Patients and Clinical Samples

This investigation enrolled 15 pathologically confirmed PDAC patients who underwent gemcitabine chemotherapy at The First Affiliated Hospital of Sun Yat‐sen University, and systematically stratified them into two cohorts based on tumor recurrence within six months: a chemotherapy‐sensitive subgroup (9/15) and a chemotherapy‐resistant subgroup (6/15).

### Establishment of Gemcitabine‐Resistant Cell Lines

Two gemcitabine‐resistant PDAC cell lines, BxPC‐3(GR) and CFPAC‐1(GR), were established via an intermittent pulsed induction protocol spanning six months. The protocol initiated with gemcitabine exposure at 100 nM in complete culture medium for 24 h, after which the drug‐containing medium was removed, cells were washed twice with PBS, and replenished with fresh gemcitabine‐free medium. Gemcitabine rechallenge cycles resumed only upon confirmation of restored cellular homeostasis, assessed through daily monitoring of morphology and proliferation kinetics. Subsequent dose escalation to 200 nM was implemented once cells exhibited unaltered growth and viability under preceding concentrations, with iterative cycles progressing until stable resistance to 1000 nM gemcitabine was achieved. Resistance was defined by sustained proliferation capacity and morphological integrity under maximum‐dose exposure, completing the adaptive gemcitabine chemoresistance induction process.

### Immunohistochemistry

Xenograft tumors derived from nude mice and PDAC clinical specimens were fixed in 4% formaldehyde. All tissue processing steps—including paraffin embedding, sectioning, and mounting on glass slides—were performed by Servicebio (Wuhan, China) under standardized histopathological protocols. Immunohistochemical detection was conducted using a 3,3′‐diaminobenzidine (DAB) kit (Servicebio) with anti‐rabbit IgG HRP‐conjugated secondary antibodies. Following DAB chromogenic development, nuclear counterstaining was achieved through hematoxylin. Two experienced observers were blinded to evaluate the staining independently, using a validated semi‐quantitative scoring system. The composite histoscore was calculated by multiplying the staining intensity grade (negative, 0; mild, 1; moderate, 2; severe, 3) by the percentage of positively stained tumor area (≤ 25%, 1; > 25% and ≤ 50%, 2; >50% and ≤ 75%, 3; >75%, 4). IF scores were measured by fluorescence intensity with ImageJ.

### mRFP‐GFP‐LC3 Puncta Assay

The autophagy flux was assessed using the mRFP‐GFP‐LC3 dual fluorescence system. Cells were infected with mRFP‐GFP‐LC3 lentivirus (Miaoling Biotechnology, Wuhan) and subsequently imaged using a Zeiss LSM780 confocal microscope. This methodology exploits the pH‐sensitive differential stability of fluorescent tags; GFP signals are quenched in the acidic lysosomal environment, whereas mRFP fluorescence remains stable across lysosomal pH gradients. Autophagic flux was quantified by fluorescence signatures—yellow puncta and red puncta. A progressive increase in both yellow and red puncta densities served as a validated metric for enhanced autophagy flux.

### Animal Experiments

### Animal Experiments—Establishment of Gemcitabine‐Resistant PDX

To establish a gemcitabine‐resistant PDX model, fresh tumor specimens from pathologically confirmed PDAC patients were surgically resected and processed into 3 mm^3^ tissue fragments. These fragments were subsequently engrafted into the axillary subcutaneous tissue of 6‐week‐old female BALB/c‐nude mice (Laboratory Animal Center of Sun Yat‐Sen University) under aseptic conditions. Following successful engraftment, first‐generation (P1) tumors were harvested upon reaching 1 cm^3^ in volume and serially passaged to establish second‐generation (P2) PDX models. The established PDX (P2) tumors were then propagated through three consecutive generations in nude mice (P3, P4, P5). When the tumor reached 4 × 4 × 4 mm^3^, mice received either gemcitabine (50 mg kg^−1^, intraperitoneal injection, twice a week) or PBS as a control during passages 3‐5 (P3‐P5). It was found that PDX presented insensitivity to gemcitabine after three consecutive passages under the treatment of gemcitabine. Then, the P5 PDX tumor exposed to gemcitabine treatment was defined as the gemcitabine‐resistant PDX model.

### Animal Experiments—Cell Line‐Derived Xenograft Models

Subcutaneous cell line‐derived xenograft models were established by inoculating gemcitabine‐resistant PDAC cells (5 million cells/100 µL PBS) into the right flank of 6‐week‐old female nude mice. Following 7 days of tumor engraftment, mice were randomized into two groups: PBS or gemcitabine (50 mg kg^−1^, intraperitoneally, twice a week).

### Animal Experiments—Drug Combination Experiment

PDX tumors were transplanted into nude mice and stratified into four groups: vehicle control, gemcitabine (TargetMol, USA, T0251) (50 mg kg^−1^), 673A (TargetMol, USA, T71998) (20 mg kg^−1^), and gemcitabine+673A combination (intraperitoneally, twice a week, for 21 days). Tumor volumes were longitudinally monitored every 4 days using digital calipers and calculated as volume = 0.52 × length × width^2^. Upon completion of the 3‐week regimen, mice were humanely euthanized for tumor excision and subsequent experiments.

### Statistical Analysis

Data were presented as mean ± standard deviation (SD). The sample size for each statistical analysis was n ≥3. The statistical method used to assess significant differences was Student's *t*‐test (two‐tailed). Correlations between variables were evaluated by Spearman's correlation coefficient. A threshold of *p* < 0.05 was predefined as statistically significant for all hypothesis tests. Statistical analyses were performed by GraphPad Prism 9.5.

### Ethics Approval Statement

Collection of clinical samples strictly complied with the Declaration of Helsinki and received formal ethical approval from the Clinical Research and Laboratory Animal Ethics Committee of the First Affiliated Hospital of Sun Yat‐sen University (Ethics No. [2023]021). Written informed consents were obtained from all patients.

All animal studies were conducted in strict adherence to the ARRIVE guidelines and approved by the Clinical Research and Laboratory Animal Ethics Committee of the First Affiliated Hospital of Sun Yat‐sen University (Ethics No. [2023]029).

## Conflict of Interest

The authors declare no conflict of interest.

## Author Contributions

X.‐T.H., J.C., E.‐L.Z., and M.‐J.M. contributed equally to this study. X.‐Y.Y. conceived and designed the study. X.‐T.H., J.C., E.‐L.Z., and M.‐J.M. performed experiments. X.‐T.H., J.C., E.‐L.Z., M.‐J.M., Y.‐Y.‐H.Y., Y.‐Q.Z., and J.‐Y.Y. analyzed the data. X.‐T.H., J.C., E.‐L.Z., M.‐J.M., J.‐Z.X., and Z.‐Y.Z. assisted in the collection and analysis of clinical samples. X.‐Y.Y., X.‐T.H., J.C., E.‐L.Z., and M.‐J.M. prepared the manuscript. X.‐Y.Y. supervised the study and reviewed the manuscript. All authors read and approved the final version of the manuscript.

## Supporting information



Supporting Information

## Data Availability

The data that support the findings of this study are available from the corresponding author upon reasonable request.
