# Supplementary material for: Positive Feedback Loop of Histone Lactylation‐Driven HNRNPC Promotes Autophagy to Confer Pancreatic Ductal Adenocarcinoma Gemcitabine Resistance
Source: Adv Sci (Weinh). 2025 Nov 27;13(8):e10483. doi: 10.1002/advs.202510483 (PMC12884808; doi:10.1002/advs.202510483)
Supplement: Supplementary file 1 — Supporting Information [file ADVS-13-e10483-s001.docx]

**Supplemental Materials and Methods**

**Cell culture and transfection**

The human PDAC cell lines BxPC-3 and CFPAC-1 were obtained from Cellcook Biotech Co., Ltd. (Guangzhou, China). Cells were cultured in RPMI 1640 medium (Gibco, USA) supplemented with 10% fetal bovine serum (Gibco, USA) and maintained at 37°C in a humidified atmosphere containing 5% CO_2_ using a Thermo Fisher Scientific incubator (USA)

Plasmid mRFP-EGFP-LC3, TRAF6, shHNRNPC and shTRAF6 were purchased from MiaolingBio (Wuhan, China).

**RNA extraction and quantitative real-time PCR**

Total RNA was isolated from distinct cellular groups using the RNA isolater Total RNA Extraction Reagent (Vazyme, Nanjing, China) in strict accordance with the manufacturer's protocol. Following RNA extraction, cDNA synthesis was performed with ABScript Neo RT Master Mix for qPCR with gDNA Remover (ABclonal, Wuhan, China) under standardized reverse transcription conditions. Quantitative gene expression analysis was subsequently carried out through real-time PCR (RT-qPCR) employing BrightCycle Universal SYBR Green qPCR Mix with UDG (ABclonal, Wuhan, China) on the QuantStudio 6 Flex Real-Time PCR System (Applied Biosystems, USA). Relative mRNA expression levels were calculated using the comparative threshold cycle (2^−ΔΔCT^) method with three technical replicates.

**Western blot**

Total proteins were extracted from cells subjected to distinct experimental conditions using a lysis buffer (BeyoTime, Shanghai, China) supplemented with a protease inhibitor cocktail (TOPSCIENCE, Shanghai, China). Protein concentrations were determined via BCA assay (Thermo Fisher, USA), followed by denaturation at 95°C for 10 min in loading buffer. Electrophoretic separation was performed using precast FuturePAGE gels (ACE Biotechnology, Nanjing, China). Resolved proteins were subsequently transferred onto polyvinylidene difluoride (PVDF) membranes via wet transfer methodology. Membranes were blocked with 5% skim milk dissolved in Tris-buffered saline containing 0.1% Tween-20 (TBST) for 1 h at room temperature. Primary antibodies were incubated with membranes overnight at 4°C under gentle rocking. Following three sequential washes with TBST buffer (10 min/wash), membranes were incubated with horseradish peroxidase (HRP)-conjugated secondary antibodies for 1 h at room temperature. Protein bands were detected using an enhanced chemiluminescent kit (New Cell & Molecular Biotech, Suzhou, China).

**Cell counting kit-8 assay**

Cell viability was assessed using the Cell Counting Kit-8 (ShareBio, Shanghai, China). PDAC cells were plated in 96-well microplates at a density of 2 × 10³ cells per well and allowed to adhere for 24 hours under standard culture conditions (37°C, 5% CO₂), and different treatments were applied to PDAC resistant cells. The optical density (OD) values exhibited a positive correlation with viable cell counts, enabling interpretation of cellular proliferation.

**Apoptosis assay**

PDAC cells under different treatment were plated in six-well plates and maintained for 48 h prior to apoptosis assessment. Following the manufacturer's protocol (LiankeBio, Hangzhou, China), cells were harvested and dual-stained with Annexin V-APC/7-AAD using the apoptosis detection kit. Quantitative analysis of apoptotic rates was subsequently performed using a flow cytometer under standardized instrument settings.

**TUNEL Assay**

TUNEL assay was performed on paraffin-embedded tissue sections by using Fluorescein (FITC) Tunel Cell Apoptosis Detection Kit (Servicebio, Wuhan, China) following the manufacturer's standardized protocol, with results expressed by the number of TUNEL-positive cells in the field of view to represent the apoptosis of the tissues.

**Chromatin Immunoprecipitation (ChIP)**

Following protein-DNA crosslinking (1% formaldehyde, 10 min) and quenching with 250 mM glycine, chromatin was fragmented using the SimpleChIP Enzymatic Chromatin IP Kit (Cell Signaling Technology) according to manufacturer specifications. Immunoprecipitation was performed with anti-H3K18La antibody. Post-IP, ChIP DNA was isolated and purified using a PCR Clean Up/DNA Purification Kit (Beyotime, Shanghai, China). Purified DNA was subsequently subjected to sequencing or qPCR analysis.

**RNA Immunoprecipitation (RIP)**

PDAC cells were lysed in RIP buffer. Lysates were subjected to immunoprecipitation using anti-HNRNPC or anti-m6A antibodies conjugated to magnetic beads. Post-immunoprecipitation, RNA-protein complexes were treated with proteinase K, and total RNA was isolated using the RNA Isolater Total RNA Extraction Reagent (Vazyme, Nanjing, China). Purified RNA was subsequently analyzed by high-throughput sequencing or qPCR.

**RNA decay assays**

PDAC cells were treated with 5 μg/ml actinomycin D (Sigma-Aldrich, USA) for 0, 1, or 3 h to inhibit transcription. Total RNA was subsequently isolated using RNA Isolater Total RNA Extraction Reagent (Vazyme, Nanjing, China). RNA stability was assessed by qPCR to quantify transcript levels of target genes.

| 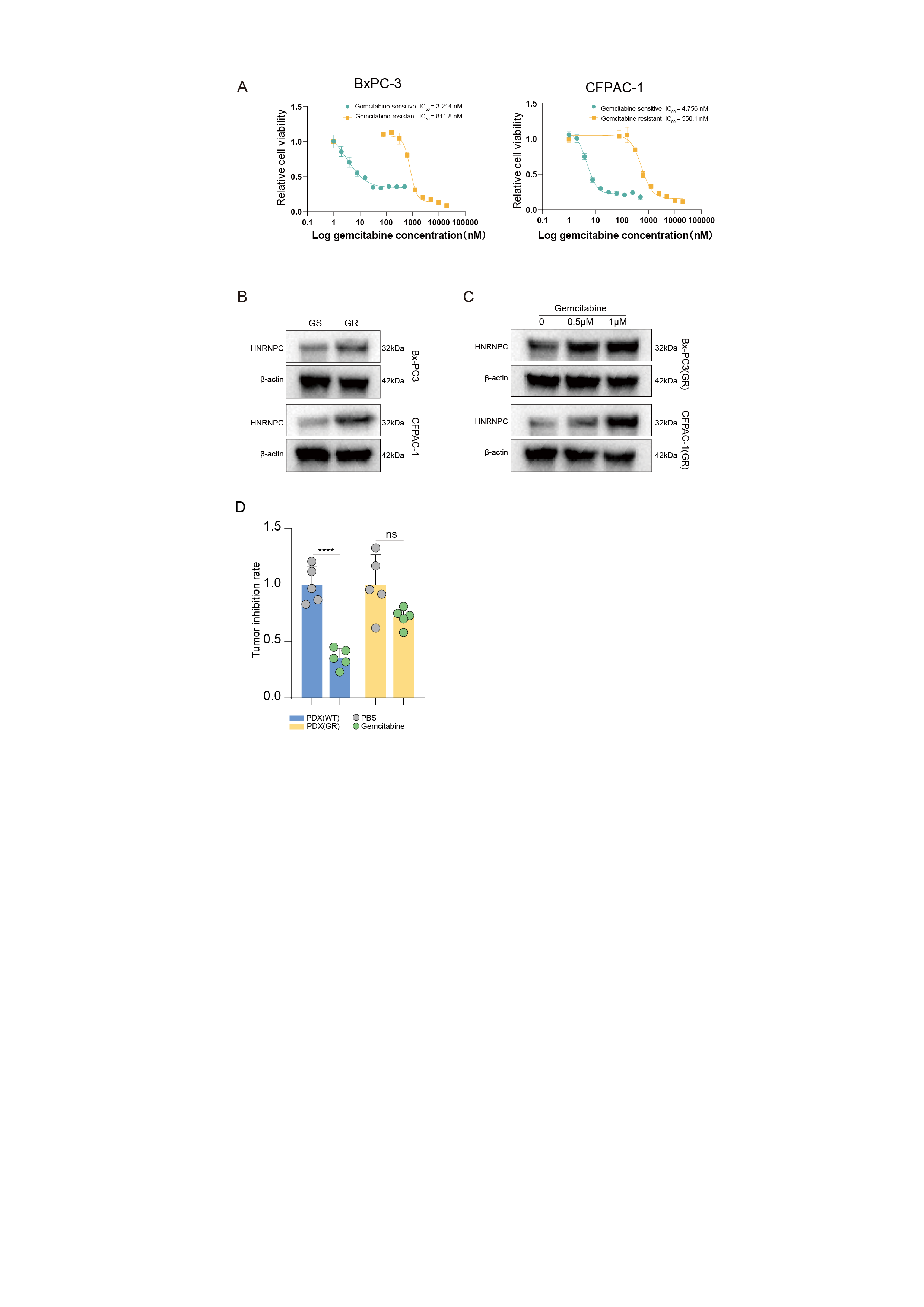 |
| --- |
| **FigureS1. HNRNPC is upregulated in gemcitabine-resistant PDAC and correlates with poor gemcitabine therapy response. A)** IC50 of gemcitabine-sensitive (GS)/gemcitabine-resistant (GR) BxPC-3 and CFPAC-1. **B)** Western blot showed up-regulated HNRNPC in BxPC-3(GR) and CFPAC-1(GR). **C)** Western blot showed gemcitabine dose-dependent up-regulated HNRNPC in BxPC-3(GR) and CFPAC-1(GR). **D)** Inhibition rate of gemcitabine on PDX tumors. WT, wild type; GR, gemcitabine resistant. |

| 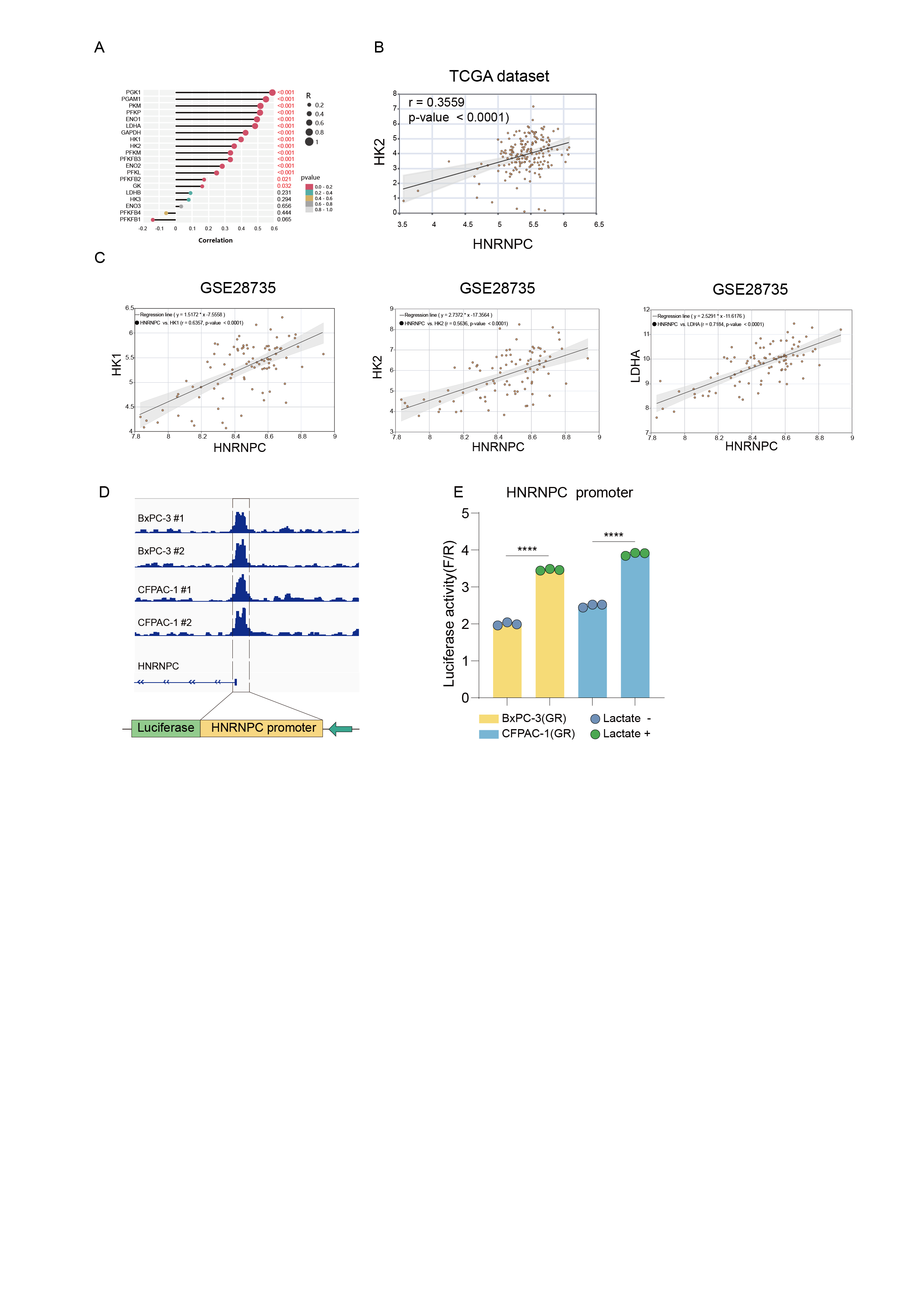 |
| --- |
| **FigureS2. HNRNPC expression is activated by histone lactylation. A)** Correlation analysis between HNRNPC and glycolysis enzymes by using TCGA dataset. **B)** Scatter plots showed the correlation between HNRNPC and HK2 analyzed with TCGA dataset. **C)** Scatter plots showed the correlation between HNRNPC and HK1/HK2/LDHA analyzed with GSE28735 dataset. **D)** Schematic diagram of the dual-luciferase structure. **E)** Luciferase activity was detected in pancreatic cancer cell with/without lactate (20mM). The data are shown as mean ± SD. *p < 0.05; ** p < 0.01; ***p < 0.001 according to Student’s t-test. |

| 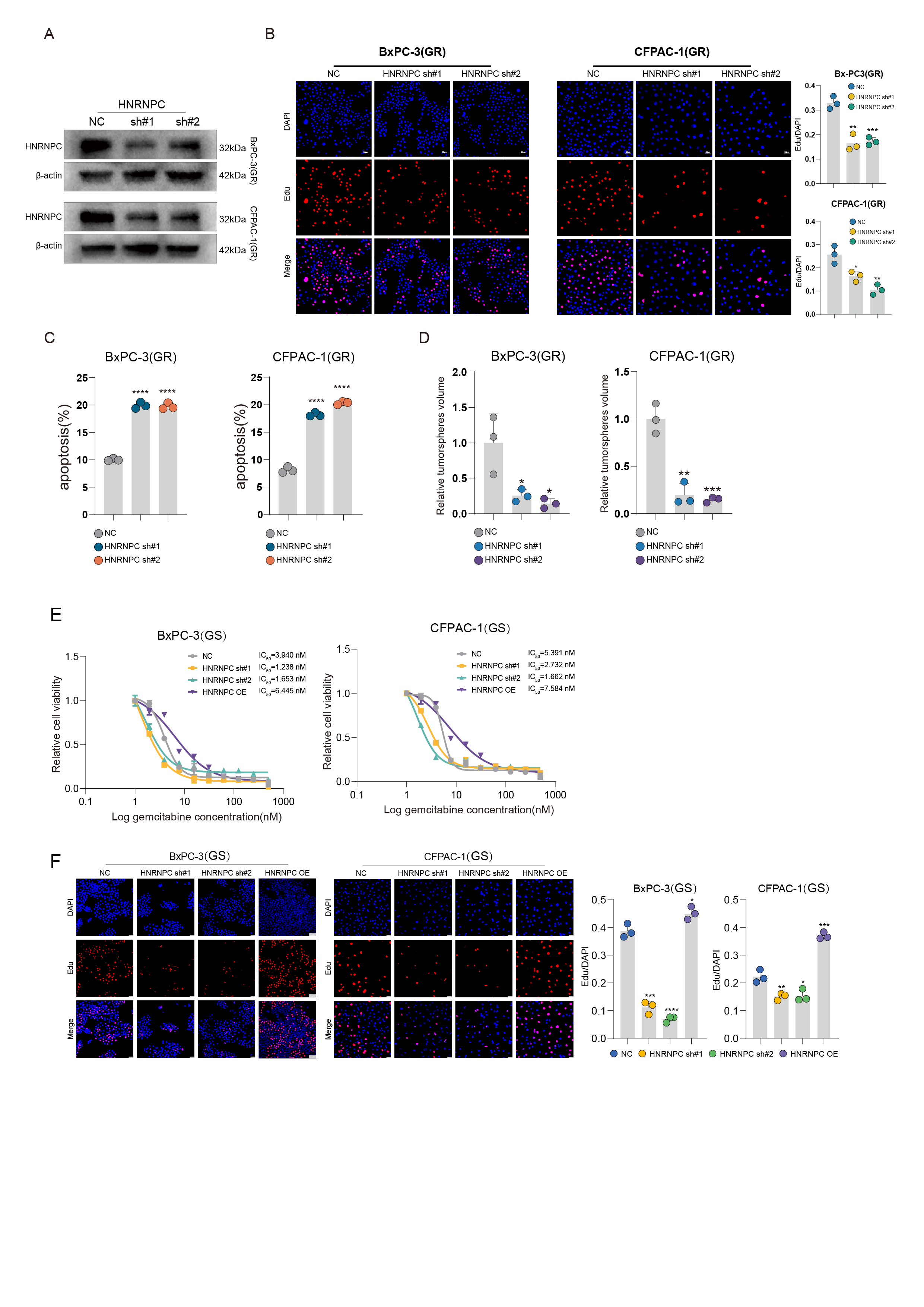 |
| --- |
| **FigureS3. HNRNPC knockdown enhances gemcitabine efficacy against PDAC in vitro and in vivo. A)** HNRNPC expression after HNRNPC knockdown detected by western blot. **B)** Edu assay in BxPC-3 (GR)/CFPAC-1 (GR) treated with 1μM gemcitabine as representative images and statistical analysis were displayed. **C)** Statistical analysis of apoptosis assay in BxPC-3 (GR)/CFPAC-1 (GR) treated with 1μM gemcitabine. **D)** Statistical analysis of tumor spheres formation in BxPC-3 (GR)/CFPAC-1 (GR) treated with 1μM gemcitabine. **E)** Pancreatic cancer cell IC50. **F)** Proliferation activity of pancreatic cancer detected by Edu and related statistical charts. The data are shown as mean ± SD. *p < 0.05; ** p < 0.01; ***p < 0.001 according to Student’s t-test. |

| 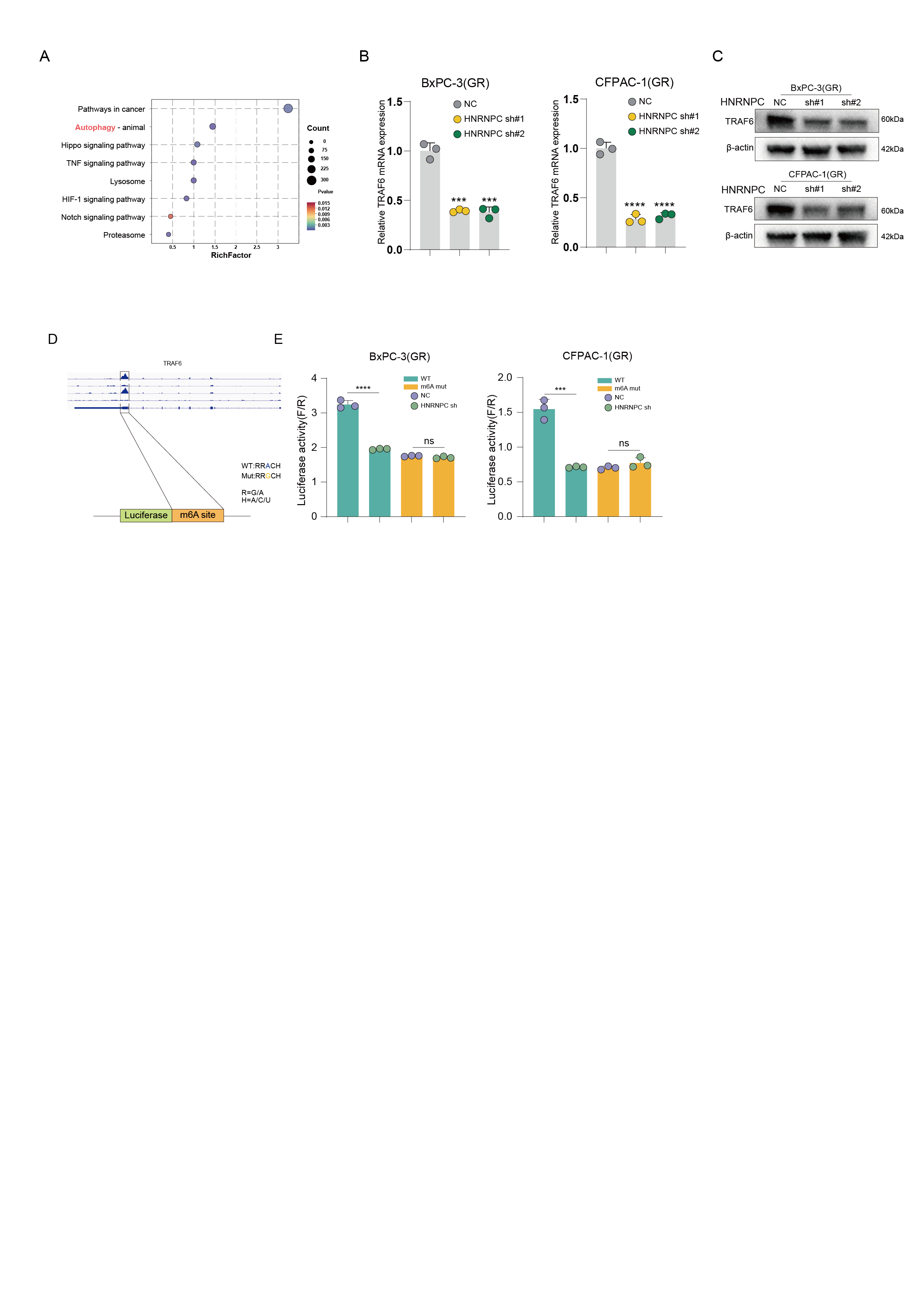 |
| --- |
| **FigureS4. HNRNPC activates autophagy by stabilizing TRAF6 mRNA through m6A-dependent mechanisms in PDAC.** **A)** KEGG enrichment analysis of HNRNPC knockdown differential genes in PDAC cells. **B)** TRAF6 expression in HNRNPC knockdown PDAC cells evaluated by qPCR. **C)** TRAF6 expression in HNRNPC knockdown PDAC cells evaluated by western blot. **D)** Schematic diagram of the dual-luciferase structure. **E)** The luciferase activity of dual-luciferase along with the TRAF6 m6A modification sequence mutation and/or HNRNPC knockdown in pancreatic cancer cells. The data are shown as mean ± SD. *p < 0.05; ** p < 0.01; ***p < 0.001 according to Student’s t-test. |

| 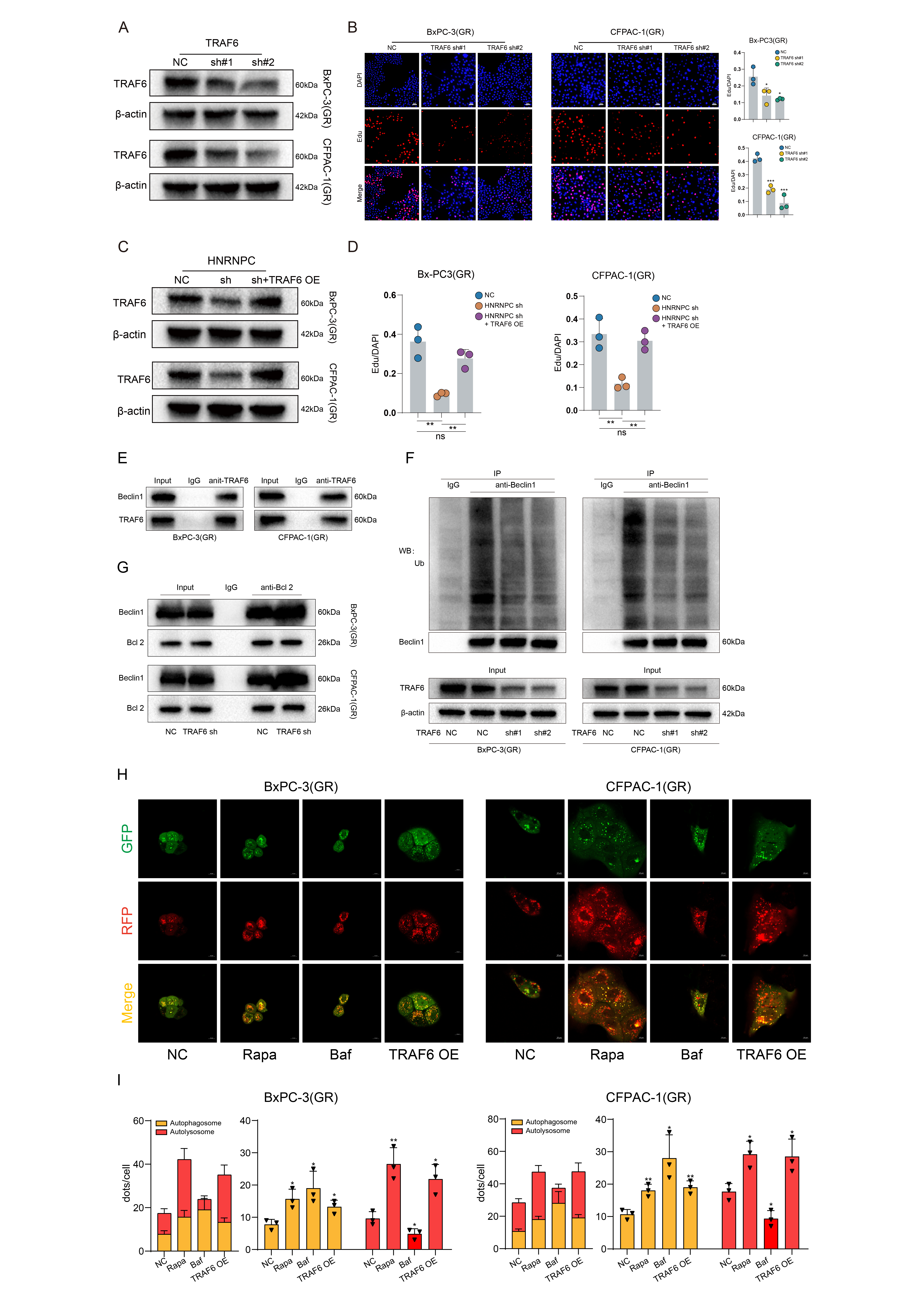 |
| --- |
| **FigureS5. TRAF6 is responsible for HNRNPC-mediated gemcitabine resistance in PDAC. A)** TRAF6 expression in TRAF6 knockdown PDAC cells evaluated by western blot. **B)** Edu assay in BxPC-3 (GR)/CFPAC-1 (GR) treated with 1μM gemcitabine as representative images and statistical analysis were displayed. **C)** TRAF6 expression in TRAF6 overexpression PDAC cells evaluated by western blot. **D)** Statistical analysis of Edu assay in PDAC cells. **E)** Co-immunoprecipitation of TRAF6 and Beclin1. **F)** Western blot was used to detect the ubiquitination modification of Beclin1 after TRAF6 knockdown. **G)** Co-immunoprecipitation of Bcl2 and Beclin1 after TRAF6 knockdown. **H)** Representative images of autophagy formation monitored by mRFP-GFP-LC3 puncta assay. **I)** Statistical analysis of RFP-GFP-LC3 puncta assay. The data are shown as mean ± SD. *p < 0.05; ** p < 0.01; ***p < 0.001 according to Student’s t-test. |

| 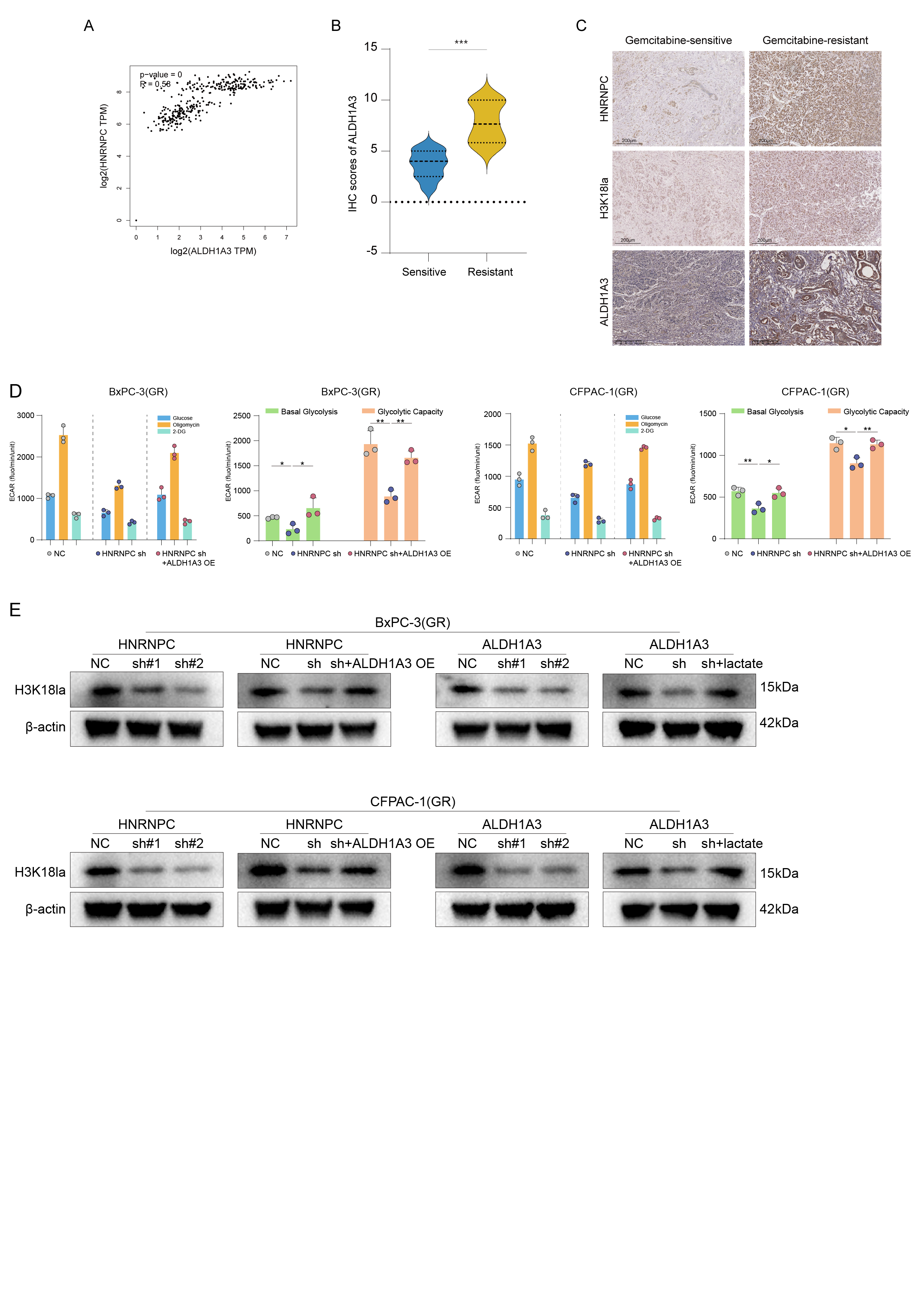 |
| --- |
| **FigureS6. HNRNPC maintains ALDH1A3 mRNA stability reinforcing H3K18la to establish a positive feedback loop. A)** Correlation analysis between ALDH1A3 and HNRNPC in TCGA dataset. **B)** Representative images of IHC staining for ALDH1A3, H3K18la, HNRNPC in PDAC patients. **C)** Statistical analysis of IHC staining for ALDH1A3 in PDAC patients. **D)** Extracellular Acidification Rate (ECAR) was used to detect the glycolytic level of pancreatic cancer cells. **E)** Western blot was used to detect the H3K18la level in pancreatic cancer cells. The data are shown as mean ± SD. *p < 0.05; ** p < 0.01; ***p < 0.001 according to Student’s t-test. |

| **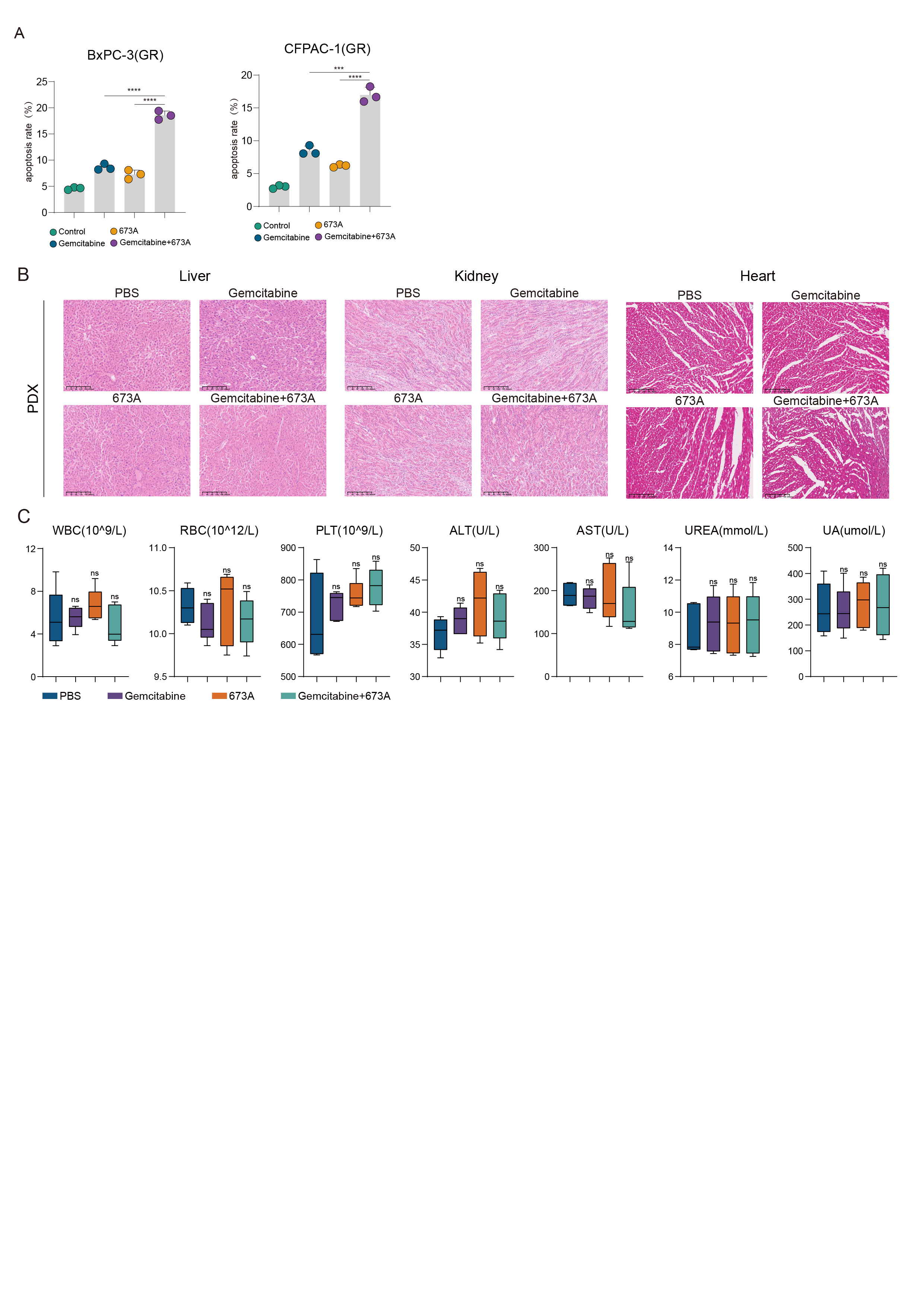** |
| --- |
| **FigureS7. Pharmaceutical 673A targets feedback signal to potentiate gemcitabine efficacy against gemcitabine-resistant PDAC. A)** Statistical analysis of apoptosis assay in BxPC-3 (GR)/CFPAC-1 (GR). The data are shown as mean ± SD. *p < 0.05; ** p < 0.01; ***p < 0.001 according to Student’s t-test. **B)** HE staining of liver, kidney and heart tissues of PDX. **C)** PDX blood test. |

**Supplementary Tables**

**Table S1. Primers Used in this Study**

| **Primer** |  | **5’-3’ Sequence** |
| --- | --- | --- |
| HNRNPC | F | GGAGATGTACGGGTCAGTAACA |
|  | R | CCCGAGCAATAGGAGGAGGA |
| TRAF6 | F | ATGCGGCCATAGGTTCTGC |
|  | R | TCCTCAAGATGTCTCAGTTCCAT |
| ALDH1A3 | F | TGAATGGCACGAATCCAAGAG |
|  | R | CACGTCGGGCTTATCTCCT |
| β-actin | F | CCTGGCACCCAGCACAAT |
|  | R | GGGCCGGACTCGTCATAC |
| HNRNPC(ChIP) | F | AACTAACCGGGCCCTCATTT |
|  | R | CATTTTCCCTGGCGATCGTG |
| TRAF6（RIP） | F | GAGGGTTTTCAGCCACGAAG |
|  | R | ACAAGGCGGTAGTGATTTTCA |
| ALDH1A3（RIP） | F | GACCGTGAGATTCGGCTTCA |
|  | R | TTTGCTATGCTGTTGTGGCG |

**Table S2. Antibodies Used in this Study**

| **Antibody** | **Catalog Number** | **Brand** |
| --- | --- | --- |
| HNRNPC | 11760-1-AP | Proteintech |
| H3K18la | 1427RM | PTMBIO |
| m6A | ab151230 | abcam |
| TRAF6 | A23385 | ABclonal |
| ALDH1A3 | 25167-1-AP | Proteintech |
| LC3 | 14600-1-AP | Proteintech |
| β-actin | GB12001-100 | Servicebio |
| Ki67 | ab156956 | abcam |
| HRP-anti-rabbit IgG | GB23303 | Servicebio |
| HRP-anti-mouse IgG | GB23301 | Servicebio |
